# Genetic Characterization of Avian Influenza Viruses Isolated from the Izumi Plain, Japan in 2019/20 Winter Season

**DOI:** 10.3390/pathogens11091013

**Published:** 2022-09-05

**Authors:** Kosuke Okuya, Ahmed Magdy Khalil, Mana Esaki, Isshu Kojima, Natsuko Nishi, Donna Koyamada, Tsutomu Matsui, Yuuhei Yoshida, Makoto Ozawa

**Affiliations:** 1 Department of Pathogenetic and Preventive Veterinary Science, Joint Faculty of Veterinary Medicine, Kagoshima University, Kagoshima 890-0065, Japan; 2 Joint Graduate School of Veterinary Medicine, Kagoshima University, Kagoshima 890-0065, Japan; 3 Transboundary Animal Diseases Research Center, Joint Faculty of Veterinary Medicine, Kagoshima University, Kagoshima 890-0065, Japan; 4 Department of Zoonotic Diseases, Faculty of Veterinary Medicine, Zagazig University, Zagazig 44511, Egypt; 5 United Graduate School of Veterinary Science, Yamaguchi University, Yamaguchi 753-8511, Japan; 6 Kagoshima Crane Conservation Committee, Izumi, Kagoshima 899-0208, Japan; 7 Matsuoka Research Institute for Science, Tokyo 108-0014, Japan

**Keywords:** avian influenza viruses, environmental water, genetic constellations

## Abstract

The Izumi plain in the Kagoshima Prefecture, Japan, is known as an overwintering site for more than 30,000 migratory waterfowl, including endangered crane species. We previously reported that environmental water samples, from artificial wet paddies created as crane roost sites on the Izumi plain, are useful for avian influenza virus (AIV) surveillance. During the 2019/20 winter season, we collected 238 water samples from the crane roost sites and isolated 22 AIVs of six subtypes: one H1N1, one H3N2, seven H3N8, four H4N6, nine H6N6, and one H11N2 subtypes. Genetic analyses revealed that AIVs of the same subtype isolated from the Izumi plain during a single winter season exhibited multiple genetic constellations. Furthermore, phylogenetic analyses suggested that our H3N2 isolate may be a genetic reassortant between close relatives to our H3N8 and H11N2 isolates. Our study highlighted the importance of monitoring AIV circulation to better understand AIV ecology in migratory waterfowl populations.

## 1. Introduction

Influenza A viruses, members of the family Orthomyxoviridae, have eight-segmented RNA genomes. Based on the antigenicity of two surface glycoproteins, hemagglutinin (HA) and neuraminidase (NA), avian influenza viruses (AIVs) are classified into 16 HA and 9 NA subtypes [1]. Because of the segmented viral genome of AIVs, co-infecting a single cell with multiple virus strains potentially results in exchanging individual gene segments with each other [2,3]. This genetic reassortment allows AIVs to evolve rapidly with the emergence of genetic variants [4,5].

The Izumi plain in the Kagoshima Prefecture, Japan, is known to be an overwintering site for over 10,000 endangered cranes, including the white-naped crane (*Grus vipio*) and the hooded crane (*Grus monacha*). To protect these endangered cranes, artificial wet paddies are created as crane roost sites during the winter season on the Izumi plain. In addition to these cranes, many other migratory waterfowl, including more than 20,000 wild ducks, visit the Izumi plain every winter. Importantly, migratory waterfowl, especially wild ducks of the orders Anseriformes and Charadriiformes, are known as natural reservoirs of AIVs [1,6]. Therefore, the Izumi plain is at potential risk of AIV invasion. 

In a previous study, we established a method for isolating AIVs from water samples collected from crane roost sites on the Izumi plain [7]. Using this method, we isolated various AIVs, including high pathogenicity avian influenza viruses (HPAIVs), from the environmental water on the Izumi plain, almost every winter season [7,8,9,10,11]. More recently, we reported an improved method for AIV isolation from environmental water samples in terms of the isolation efficiency [12]. In the improved AIV isolation method, potential viruses in environmental water samples were concentrated by adsorption to chicken red blood cells (RBCs) and inoculated into two embryonated chicken eggs per sample. This improved method enabled efficient AIV isolation from the crane roost site water samples (44/234, 18.8%) during the 2018/19 winter season. However, the subtypes of 17 of the 44 isolates (38.6%) were confirmed to be mixed [12]. These results suggest that these 17 AIVs were isolated by inoculating multiple AIV strains of various subtypes contained in the environmental water samples into single eggs. Because of the segmented genome nature, genetic constellations of AIVs of mixed subtypes are difficult to determine. In this study, to improve the possibility of single AIV isolation, we increased the number of embryonated chicken eggs used for inoculating samples from two to four per sample, resulting in the two-fold dilution of the inoculums. By applying this improved AIV isolation method to AIV surveillance on the Izumi plain during the 2019/20 winter season, we revealed that AIVs of the same subtype isolated from the Izumi plain during a single winter season exhibited multiple genetic constellations.

## 2. Results

### 2.1. AIV Isolation from Crane Roost Site Water on the Izumi Plain during the 2019/20 Winter Season

We isolated 23 AIVs from 238 crane roost site water samples on the Izumi plain during the 2019/20 winter season (Table 1). Notably, most AIVs were isolated at the beginning of winter (18 November to 23 December). PCR-based HA and NA subtyping was applied to determine the HA and NA subtypes of our 23 AIV isolates. The HA subtype of one of the 23 isolates was mixed with H4 and H6 gene segments. The remaining 22 isolates included six subtypes: one H1N1, one H3N2, seven H3N8, four H4N6, eight H6N6, and one H11N2 subtypes (Table 1). Sequence analysis of the HA gene segment revealed that all 22 isolates had monomeric basic residues at the HA cleavage site, suggesting limited pathogenicity in chickens.

### 2.2. Phylogenetic Analyses of Our 22 AIV Isolates

**Figure 1:**
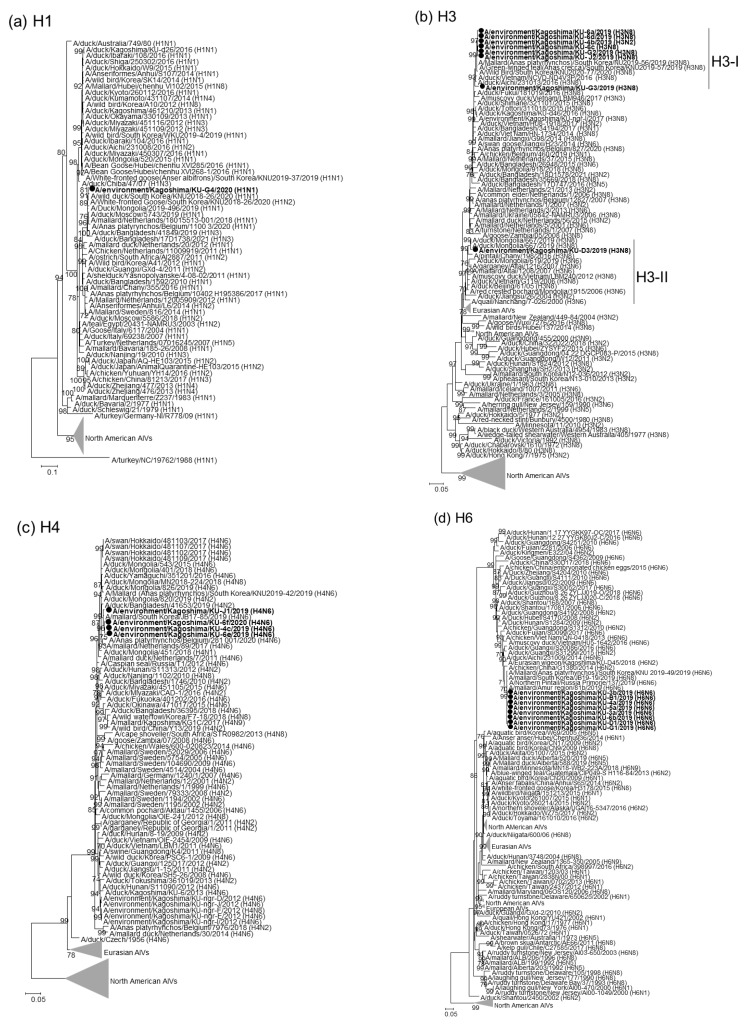

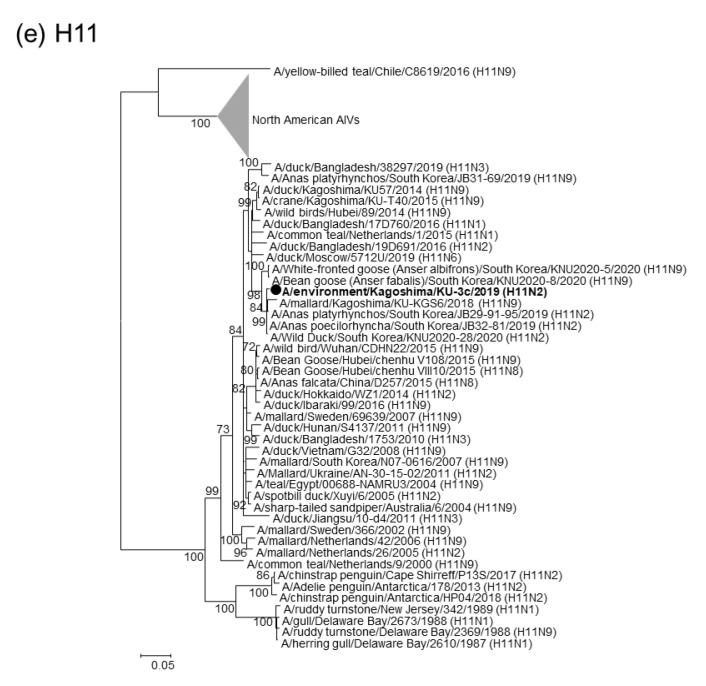
Phylogenetic trees of the HA genes. The 22 AIVs isolated in this study are indicated by black circles in the phylogenetic trees of H1 (**a**), H3 (**b**), H4 (**c**), H6 (**d**), and H11 (**e**) HA genes. Our isolates were phylogenetically analyzed with their representative counterparts using the maximum-likelihood method with a bootstrapping set of 1000 replicates. Bootstrap values of >70% are shown at the nodes. The scale bar indicates the number of nucleotide substitutions per site.

Nucleotide sequences for individual gene segments of the 22 AIV isolates were determined and phylogenetically analyzed with representative AIV strains that reflect the temporal and spatial distribution. The phylogenetic tree for the H1 HA gene (Figure 1a) demonstrated that the HA gene segment from A/environment/Kagoshima/KU-G4/2020 (H1N1) was phylogenetically close to those of the H1N1 and H1N2 AIVs isolated from wild ducks, domestic ducks, and chickens in Asian and European countries. The H3 gene segments from our H3 isolates clustered with Asian isolates in two genetic clades and were tentatively identified as clades H3-I and H3-II (Figure 1b). Seven of our eight H3 isolates were categorized as clade H3-I, while the remaining H3 isolate, A/environment/Kagoshima/KU-D3/2019 (H3N8), fell into clade H3-II. The H4 and H6 HA gene segments from our four H4N6 and eight H6N6 isolates formed single clusters in the phylogenetic trees for each HA gene (Figure 1c,d). The H11 HA gene segment from A/environment/Kagoshima/KU-3c/2019 (H11N2) was phylogenetically close to those from H11N2 AIVs reported from South Korea in 2019 and from an H11N9 AIV previously isolated from a mallard on the Izumi plain, A/mallard/Kagoshima/KU-KGS6/2018 (H11N9) (Figure 1e).

**Figure 2:**
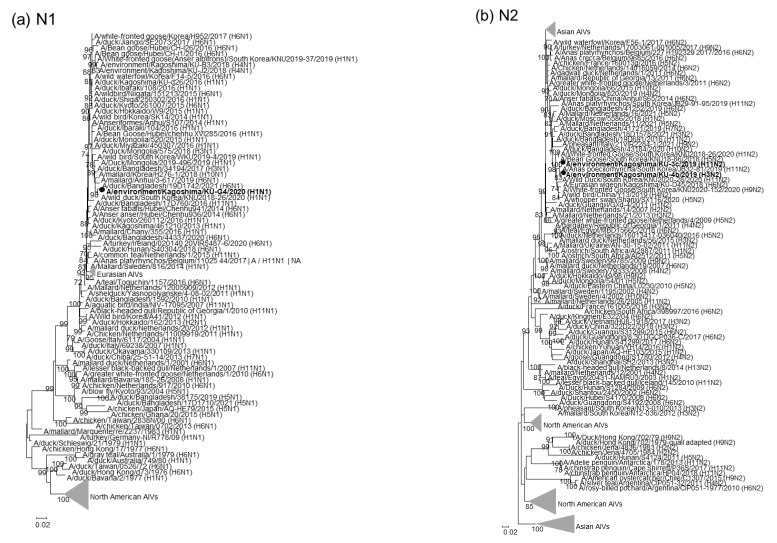

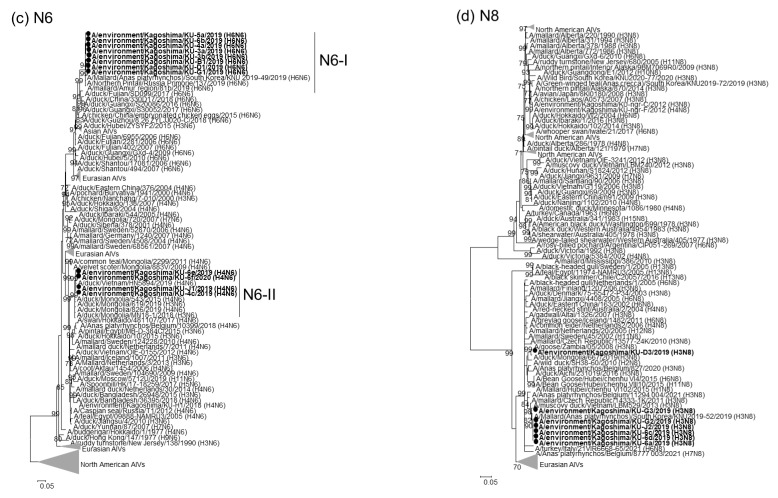
Phylogenetic trees of the NA genes. The 22 AIVs isolated in this study are indicated by black circles in the phylogenetic trees of N1 (**a**), N2 (**b**), N6 (**c**), and N8 (**d**) NA genes. Our isolates were phylogenetically analyzed with their representative counterparts using the maximum-likelihood method with a bootstrapping set of 1000 replicates. Bootstrap values of >70% are shown at the nodes. The scale bar indicates the number of nucleotide substitutions per site.

Phylogenetic trees for the NA gene segments revealed that our H1N1 isolate-derived N1 NA gene segments formed a cluster with those from the Asian isolates (Figure 2a). Intriguingly, our H3N2 and H11N2 isolate-derived N2 NA gene segments formed a single cluster with their counterparts from H11N2 AIVs recently isolated in South Korea (Figure 2b), suggesting that these isolates of different subtypes shared a common ancestor for their NA gene segments. In contrast, the N6 NA gene segments from our H4N6 and H6N6 isolates individually formed single clusters (Figure 2c), indicating that the N6 NA gene segments from our H4N6 and H6N6 isolates were phylogenetically distant. The N8 NA gene segments from all of our H3N8 isolates, except for A/environment/Kagoshima/KU-D3/2019 (H3N8), formed a single cluster (Figure 2d).

The phylogenetic analyses for the remaining six gene segments revealed that our 22 isolates exhibited broad phylogenetic variations. For example, the PB2 gene segments from our 22 isolates were divided into four genetic clades (tentatively designated as clades PB2-I to -IV; Supplementary Figure S1a, Table 2). Similarly, our 22 AIVs included three genetic clades of M and NS gene segments, four genetic clades of PB1 and NP gene segments, and five genetic clades of PA (Supplementary Figure S1b–f, Table 2). Interestingly, the PB2 gene segments from AIVs of different subtypes, that is, A/environment/Kagoshima/KU-J1/2019 (H4N6), A/environment/Kagoshima/KU-6d/2019 (H3N8), A/environment/Kagoshima/KU-4b/2019 (H3N2), A/environment/Kagoshima/KU-6a/2019 (H3N8), A/environment/Kagoshima/KU-6c/2019 (H3N8), and A/environment/Kagoshima/KU-G2/2019 (H6N6), were categorized into the same clade, clade PB2-I (Supplementary Figure S1a, Table 2). These results indicated that the same ancestor of the PB2 gene segment is widely shared among the AIVs of different subtypes. Notably, all gene segments, except for the N2 NA gene segment, from our H3N2 isolate, A/environment/Kagoshima/KU-4b/2019 (H3N2), were almost identical to those from our three H3N8 isolates, A/environment/Kagoshima/KU-6a/2019 (H3N8), A/environment/Kagoshima/KU-6d/2019 (H3N8), and A/environment/Kagoshima/KU-6c/2019 (H3N8). Because an ancestor for the N2 NA gene segment from A/environment/Kagoshima/KU-4b/2019 (H3N2) was likely a H11N2 AIV recently circulating in East Asia (Figure 2b), these results suggested that A/environment/Kagoshima/KU-4b/2019 (H3N2) was a progeny of a genetic reassortant recently generated between H11N2 and H3N8 AIVs.

### 2.3. Genotypes of Our 22 AIV Isolates

Phylogenetic trees for each gene segment revealed that the genetic constellations of our H3N8, H4N6, and H6N6 isolates were inconsistent. The combination of the genetic clades of each gene segment allowed us to classify our H3N8, H4N6, and H6N6 isolates into five (H3N8-I to -V), three (H4N6-I to -IV), and two (H6N6-I and -II) genotypes, respectively (Table 2). These results indicated that AIVs of the same HA and NA subtypes with various genetic constellations invaded the Izumi plain during a single winter season.

## 3. Discussion

In this study, we revealed the transition of genetic constellations of AIVs among wild waterfowl overwintering on the Izumi plain during the 2019/20 winter season. We isolated 23 AIVs, including one isolate containing multiple subtypes, from 238 water samples collected from crane roost sites. The AIV-positive rate was not as high during the 2019/20 winter season (23/238, 9.7%) as it was during the previous season (44/234, 18.8%) [12]. Of the AIV-positive samples, only one isolate was confirmed to be mixed, suggesting that a two-fold dilution of the inocula by doubling the number of eggs may contribute to improved efficiency of single AIV isolation. Notably, most AIVs were isolated at the beginning of the winter season, suggesting that AIVs were actively circulating in the early winter of the 2019/20 season, similar to the previous winter seasons [7,8,9,10,11,13].

Genetic analyses revealed that the 22 AIVs isolated from crane roost site water during the 2019/20 winter season could be classified into six subtypes: H1N1, H3N2, H3N8, H4N6, H6N6, and H11N2 subtypes. As the Izumi plain is located on the East-Australian Flyway, most AIV strains invading the Izumi plain via migratory birds, especially wild ducks, during the winter season belong to the Eurasian lineage [7,10,12]. Indeed, each genetic sequence of our 22 isolates was close to the genetic sequence of Asian AIVs. Based on the genetic constellation, the H3N8, H4N6, and H6N6 isolates were divided into five, three, and two genotypes, respectively (Table 2). These results indicated that genetic reassortment among AIVs of the same and/or different subtypes occurred actively. Intriguingly, phylogenetic analyses suggested that A/environment/Kagoshima/KU-4b/2019 (H3N2) was a progeny of a genetic reassortant recently generated between H11N2 and H3N8 AIVs (Figure 1, Figure 2 and Figure S1). We cannot rule out the possibility that genetic reassortments occurred in the eggs inoculated with multiple AIV strains. However, our AIV isolates were initially subjected to PCR-based HA and NA subtyping, which enabled the detection of AIV strains of multiple subtypes in the eggs, if any were present, with a high level of sensitivity. Therefore, the genetic constellation diversity of AIVs identified in this study most likely reflect natural reassortments, rather than artificial or accidental reassortments. Overall, potential genetic reassortment among AIVs of the same and/or different subtypes may occur on the Izumi plain.

In conclusion, broad genetic variations were observed in AIVs isolated from the Izumi plain during the 2019/20 winter season. Furthermore, one isolate appears to be a reassortant emerging on the Izumi plain. Our study highlighted the importance of monitoring AIV circulation to better understand AIV ecology in migratory waterfowl populations.

## 4. Materials and Methods

### 4.1. Sample Collection

Environmental water samples (50 mL for each sample) were collected from the crane roost sites on the Izumi plain during the 2019/20 winter season. A total of 238 samples were collected; 14 samples were collected every week from November 2019 to the end of February 2020, except for the first week of January. The collected water samples were stored at approximately 4 °C until use.

### 4.2. AIV Isolation from Water Samples

AIVs were isolated from the water samples according to a previous study [12]. Briefly, environmental water samples were mixed with 10-fold concentrated phosphate-buffered saline (PBS) for osmotic adjustment and incubated with 100 µL of RBCs on ice for 1 h. The RBCs were pelleted by centrifugation (3000 rpm for 5 min at 4 °C), resuspended in 1 mL of transport medium [12] and inoculated into the allantoic cavity of ten-day-old RBCs (four eggs/sample). After incubation at 37 °C for two days, allantoic fluids were collected. AIV recovery from the collected allantoic fluids were confirmed using a hemagglutination assay, as previously described [10].

### 4.3. Real Time Reverse Transcription (RT)-PCR and HA and NA Subtyping

RNA was extracted from allantoic fluids that showed hemagglutination activity, using an InnuPREP virus DNA/RNA Kit (Analytik Jena AG, Jena, Germany). The RNA was then subjected to real time RT-PCR-based influenza A viral gene detection with an iTaq Universal SYBR Green One-Step Kit (BioRad, Hercules, CA, USA) and primer sets specific for a conserved region of influenza viral M genes [7]. The viral gene-positive RNA was reverse-transcribed using SuperScript IV Reverse Transcriptase (Thermo Fisher Scientific, Waltham, MA, USA) for cDNA synthesis. Conventional PCR-based HA and NA subtyping was performed using cDNA and Tks Gflex DNA Polymerase (TaKaRa Bio Inc., Otsu, Japan) with a panel of subtype-specific primer sets [14].

### 4.4. Sequence Analyses of AIV Genes

Nucleotide sequences of the open reading frames of all viral gene segments were determined by nanopore sequencing using the MinION Mk1b device (Oxford Nanopore Technologies, Oxford, UK), as previously described [13]. Briefly, the eight-segmented influenza viral genes were individually amplified by conventional PCR using Tks Gflex DNA Polymerase and KOD One PCR Master Mix -Blue- (TaKaRa Bio Inc.) with gene segment-specific primer sets [15] using the cDNAs synthesized above. The PCR amplicons were cleaned up and adaptor-ligated using a Direct cDNA Sequencing Kit (Oxford Nanopore Technologies) with a Native Barcoding Expansion Kit (Oxford Nanopore Technologies), and sequenced with Flongle flow cells (Oxford Nanopore Technologies) using MinION control software (Oxford Nanopore Technologies). The consensus sequences for each gene segment were generated using Geneious Prime v.2021.1.1 (Biomatters Ltd., Auckland, New Zealand). The nucleotide sequences were deposited in the Global Initiative on Sharing Avian Influenza Data (GISAID) database (http://platform.gisaid.org/) on 2 September 2022 (Table 1).

### 4.5. Phylogenetic Analyses for AIV Genes

Various AIV strains which reflect the temporal and spatial distribution were retrieved from the GISAID database. The sequences of the viral gene segments from our AIV isolates were phylogenetically analyzed with the downloaded AIVs. The nucleotide sequences were aligned using the MUSCLE program [16]. Phylogenetic trees for each viral gene were constructed using the maximum-likelihood method in MEGA 7 software [17], with a bootstrapping set of 1000 replicates. All subgroups analyzed in this study were tentatively designated based on the shape of the phylogenetic trees for each gene segment.

## Figures and Tables

**Table 1:** AIVs isolated in this study.

Subtype	Isolate	Collection Date	Isolate ID *
H1N1	A/environment/Kagoshima/KU-G4/2020 (H1N1)	27 January 2020	EPI_ISL_13374949
H3N2	A/environment/Kagoshima/KU-4b/2019 (H3N2)	2 December 2019	EPI_ISL_13374950
H3N8	A/environment/Kagoshima/KU-6a/2019 (H3N8)	18 November 2019	EPI_ISL_13374979
	A/environment/Kagoshima/KU-6c/2019 (H3N8)	25 November 2019	EPI_ISL_13375104
	A/environment/Kagoshima/KU-6d/2019 (H3N8)	25 November 2019	EPI_ISL_13375105
	A/environment/Kagoshima/KU-D3/2019 (H3N8)	2 December 2019	EPI_ISL_13375750
	A/environment/Kagoshima/KU-G2/2019 (H3N8)	2 December 2019	EPI_ISL_13375751
	A/environment/Kagoshima/KU-G3/2019 (H3N8)	2 December 2019	EPI_ISL_13417555
	A/environment/Kagoshima/KU-J2/2019 (H3N8)	9 December 2019	EPI_ISL_13418079
H4N6	A/environment/Kagoshima/KU-J1/2019 (H4N6)	25 November 2019	EPI_ISL_13418433
	A/environment/Kagoshima/KU-4c/2019 (H4N6)	23 December 2019	EPI_ISL_13418523
	A/environment/Kagoshima/KU-6e/2019 (H4N6)	23 December 2019	EPI_ISL_13418524
	A/environment/Kagoshima/KU-6f/2020 (H4N6)	20 January 2020	EPI_ISL_13418525
H6N6	A/environment/Kagoshima/KU-3a/2019 (H6N6)	18 November 2019	EPI_ISL_13437619
	A/environment/Kagoshima/KU-5a/2019 (H6N6)	18 November 2019	EPI_ISL_13437942
	A/environment/Kagoshima/KU-6b/2019 (H6N6)	18 November 2019	EPI_ISL_13437509
	A/environment/Kagoshima/KU-3b/2019 (H6N6)	25 November 2019	EPI_ISL_13439033
	A/environment/Kagoshima/KU-4a/2019 (H6N6)	25 November 2019	EPI_ISL_13439034
	A/environment/Kagoshima/KU-B1/2019 (H6N6)	25 November 2019	EPI_ISL_13439048
	A/environment/Kagoshima/KU-D1/2019 (H6N6)	25 November 2019	EPI_ISL_13439049
	A/environment/Kagoshima/KU-G1/2019 (H6N6)	25 November 2019	EPI_ISL_13439050
H11N2	A/environment/Kagoshima/KU-3c/2019 (H11N2)	16 December 2019	EPI_ISL_13439051

* Isolate IDs in the GISAID database are listed.

**Table 2:** Genetic clades of viral gene segments from 22 AIV isolates.

Subtype	Isolate	Genetic Clade * of the Following Gene Segment:								Genotype **
		HA	NA	PB2	PB1	PA	NP	M	NS	
H1N1	A/environment/Kagoshima/KU-G4/2020 (H1N1)	NA	NA	PB2-I	PB1-II	PA-I	NP-II	M-I	NS-I	NA ***
H3N2	A/environment/Kagoshima/KU-4b/2019 (H3N2)	H3-I	N2-I	PB2-I	PB1-II	PA-II	NP-I	M-II	NS-I	NA
H3N8	A/environment/Kagoshima/KU-6a/2019 (H3N8)	H3-I	N8-I	PB2-I	PB1-I	PA-II	NP-I	M-II	NS-I	H3N8-I
	A/environment/Kagoshima/KU-6c/2019 (H3N8)	H3-I	N8-I	PB2-I	PB1-II	PA-II	NP-I	M-II	NS-I	H3N8-II
	A/environment/Kagoshima/KU-6d/2019 (H3N8)	H3-I	N8-I	PB2-I	PB1-II	PA-II	NP-I	M-II	NS-I	H3N8-II
	A/environment/Kagoshima/KU-D3/2019 (H3N8)	H3-II	N8-I	PB2-II	PB1-I	PA-III	NP-IV	M-II	NS-I	H3N8-III
	A/environment/Kagoshima/KU-G2/2019 (H3N8)	H3-I	N8-I	PB2-I	PB1-II	PA-II	NP-I	M-II	NS-I	H3N8-II
	A/environment/Kagoshima/KU-G3/2019 (H3N8)	H3-I	N8-I	PB2-I	PB1-I	PA-IV	NP-II	M-I	NS-I	H3N8-IV
	A/environment/Kagoshima/KU-J2/2019 (H3N8)	H3-I	N8-I	PB2-I	PB1-III	PA-II	NP-IV	M-I	NS-I	H3N8-V
H4N6	A/environment/Kagoshima/KU-J1/2019 (H4N6)	NA	N6-II	PB2-I	PB1-II	PA-IV	NP-I	M-I	NS-I	H4N6-I
	A/environment/Kagoshima/KU-4c/2019 (H4N6)		N6-II	PB2-IV	PB1-II	PA-I	NP-IV	M-I	NS-I	H4N6-II
	A/environment/Kagoshima/KU-6e/2019 (H4N6)		N6-II	PB2-I	PB1-II	PA-II	NP-IV	M-I	NS-I	H4N6-III
	A/environment/Kagoshima/KU-6f/2020 (H4N6)		N6-II	PB2-IV	PB1-II	PA-I	NP-IV	M-I	NS-I	H4N6-II
H6N6	A/environment/Kagoshima/KU-3a/2019 (H6N6)	NA	N6-I	PB2-III	PB1-IV	PA-V	NP-III	M-III	NS-III	H6N6-I
	A/environment/Kagoshima/KU-5a/2019 (H6N6)		N6-I	PB2-III	PB1-IV	PA-V	NP-III	M-III	NS-III	H6N6-I
	A/environment/Kagoshima/KU-6b/2019 (H6N6)		N6-I	PB2-III	PB1-IV	PA-V	NP-III	M-III	NS-III	H6N6-I
	A/environment/Kagoshima/KU-3b/2019 (H6N6)		N6-I	PB2-III	PB1-IV	PA-V	NP-III	M-III	NS-III	H6N6-I
	A/environment/Kagoshima/KU-4a/2019 (H6N6)		N6-I	PB2-III	PB1-IV	PA-V	NP-III	M-III	NS-III	H6N6-I
	A/environment/Kagoshima/KU-B1/2019 (H6N6)		N6-I	PB2-III	PB1-IV	PA-V	NP-III	M-III	NS-III	H6N6-I
	A/environment/Kagoshima/KU-D1/2019 (H6N6)		N6-I	PB2-I	PB1-I	PA-II	NP-II	M-I	NS-II	H6N6-II
	A/environment/Kagoshima/KU-G1/2019 (H6N6)		N6-I	PB2-III	PB1-IV	PA-V	NP-III	M-III	NS-III	H6N6-I
H11N2	A/environment/Kagoshima/KU-3c/2019 (H11N2)	NA	N2-I	PB2-I	PB1-II	PA-III	NP-I	M-I	NS-I	NA

* Genetic clades for each viral gene segment were defined based on phylogenetic trees (Supplementary Figure S1). ** Genotypes were defined based on genetic constellations. *** NA: not applicable.

## Data Availability

The nucleotide sequences of our isolates were deposited in the Global Initiative on Sharing Avian Influenza Data (GISAID) database (http://platform.gisaid.org/) accessed on 2 September 2022.

## Supplementary Materials

The following supporting information can be downloaded at: https://www.mdpi.com/article/10.3390/pathogens11091013/s1, Figure S1: Phylogenetic trees of the remaining six genes.
