# Supplementary material for: Genetic Characterization of Avian Influenza Viruses Isolated from the Izumi Plain, Japan in 2019/20 Winter Season
Source: Pathogens. 2022 Sep 5;11(9):1013. doi: 10.3390/pathogens11091013 (PMC9505354; doi:10.3390/pathogens11091013)
Supplement: Supplementary file 1 [file pathogens-11-01013-s001.zip › Figure S1.pdf]

Supplementary Figure legends

Supplementary Figure S1. Phylogenetic trees of the remaining six genes

The 22 AIVs isolated in this study are indicated by black circles in the phylogenetic trees of PB2 (A), PB1 (B), PA (C), NP (D), M (E), and NS (F) genes. Our isolates were phylogenetically analyzed with their representative counterparts using the maximum-likelihood method with a bootstrapping set of 1,000 replicates. Bootstrap values of >70% are shown at the nodes. The scale bar indicates the number of nucleotide substitutions per site.
