# Supplementary material for: Genetic Characterization of Avian Influenza Viruses Isolated from the Izumi Plain, Japan in 2019/20 Winter Season
Source: Pathogens. 2022 Sep 5;11(9):1013. doi: 10.3390/pathogens11091013 (PMC9505354; doi:10.3390/pathogens11091013)
Supplement: Supplementary file 1 [file pathogens-11-01013-s001.zip › Figure S1.pptx]

## Slide 1
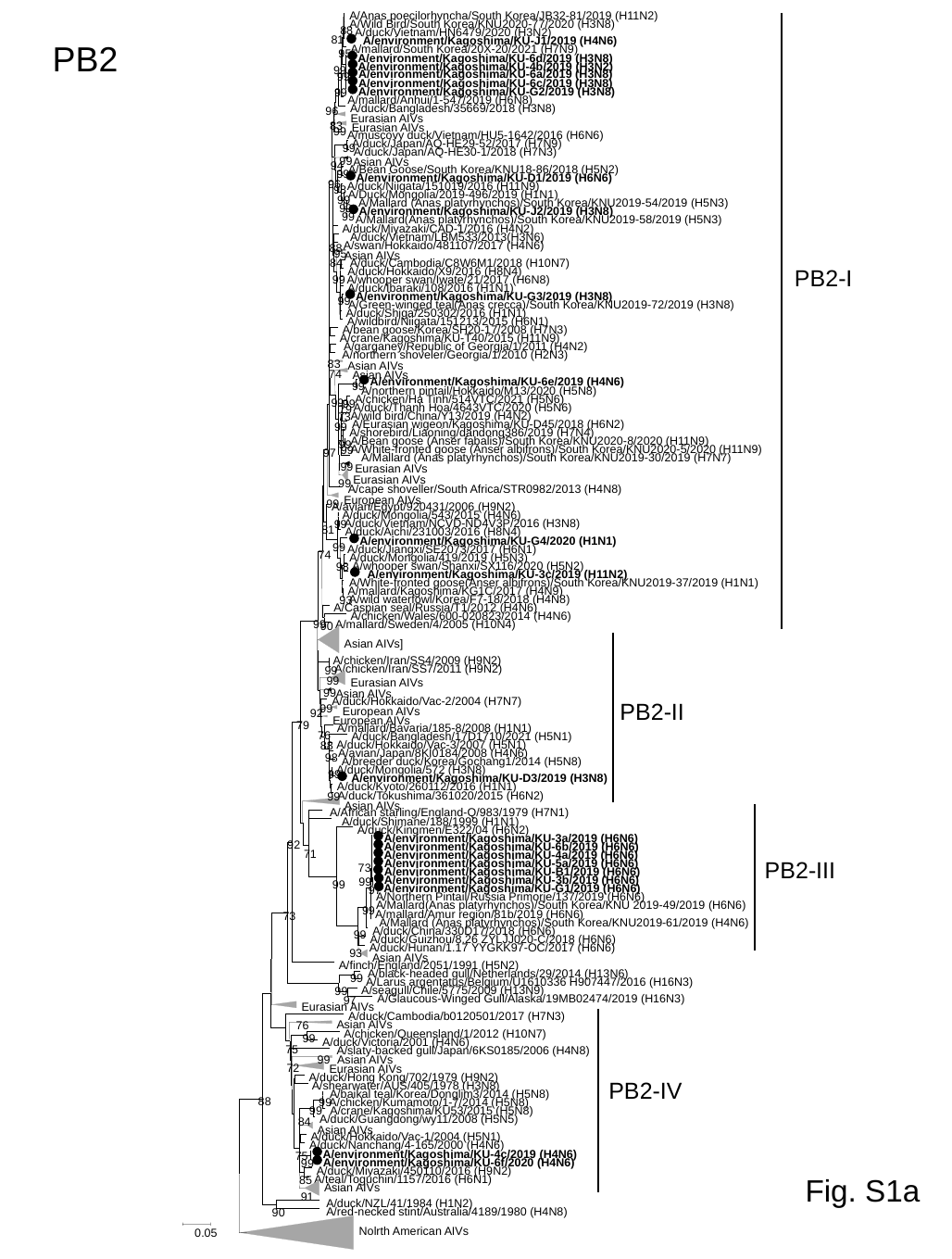

A/Anas poecilorhyncha/South Korea/JB32-81/2019 (H11N2)
 A/Wild Bird/South Korea/KNU2020-77/2020 (H3N8)
 A/duck/Vietnam/HN6479/2020 (H3N2)
 A/environment/Kagoshima/KU-J1/2019 (H4N6)
 A/mallard/South Korea/20X-20/2021 (H7N9)
 A/environment/Kagoshima/KU-6d/2019 (H3N8)
 A/environment/Kagoshima/KU-4b/2019 (H3N2)
 A/environment/Kagoshima/KU-6a/2019 (H3N8)
 A/environment/Kagoshima/KU-6c/2019 (H3N8)
 A/environment/Kagoshima/KU-G2/2019 (H3N8)
 A/mallard/Anhui/1-547/2019 (H6N8)
 A/duck/Bangladesh/35669/2018 (H3N8)
 Eurasian AIVs
 Eurasian AIVs
 A/muscovy duck/Vietnam/HU5-1642/2016 (H6N6)
 A/duck/Japan/AQ-HE29-52/2017 (H7N9)
 A/duck/Japan/AQ-HE30-1/2018 (H7N3)
 Asian AIVs
 A/Bean Goose/South Korea/KNU18-86/2018 (H5N2)
 A/environment/Kagoshima/KU-D1/2019 (H6N6)
 A/duck/Niigata/151019/2016 (H11N9)
 A/Duck/Mongolia/2019-496/2019 (H1N1)
 A/Mallard (Anas platyrhynchos)/South Korea/KNU2019-54/2019 (H5N3)
 A/environment/Kagoshima/KU-J2/2019 (H3N8)
 A/Mallard(Anas platyrhynchos)/South Korea/KNU2019-58/2019 (H5N3)
 A/duck/Miyazaki/CAD-1/2016 (H4N2)
 A/duck/Vietnam/LBM533/2013(H3N6)
 A/swan/Hokkaido/481107/2017 (H4N6)
 Asian AIVs
 A/duck/Cambodia/C8W6M1/2018 (H10N7)
 A/duck/Hokkaido/X9/2016 (H8N4)
 A/whooper swan/Iwate/21/2017 (H6N8)
 A/duck/Ibaraki/108/2016 (H1N1)
 A/environment/Kagoshima/KU-G3/2019 (H3N8)
 A/Green-winged teal(Anas crecca)/South Korea/KNU2019-72/2019 (H3N8)
 A/duck/Shiga/250302/2016 (H1N1)
 A/wildbird/Niigata/151213/2015 (H6N1)
 A/bean goose/Korea/SH20-17/2008 (H7N3)
 A/crane/Kagoshima/KU-T40/2015 (H11N9)
 A/garganey/Republic of Georgia/1/2011 (H4N2)
 A/northern shoveler/Georgia/1/2010 (H2N3)
 Asian AIVs
 Asian AIVs
 A/environment/Kagoshima/KU-6e/2019 (H4N6)
 A/northern pintail/Hokkaido/M13/2020 (H5N8)
 A/chicken/Ha Tinh/514VTC/2021 (H5N6)
 A/duck/Thanh Hoa/4643VTC/2020 (H5N6)
 A/wild bird/China/Y13/2019 (H4N2)
 A/Eurasian wigeon/Kagoshima/KU-D45/2018 (H6N2)
 A/shorebird/Liaoning/dandong386/2019 (H7N4)
 A/Bean goose (Anser fabalis)/South Korea/KNU2020-8/2020 (H11N9)
 A/White-fronted goose (Anser albifrons)/South Korea/KNU2020-5/2020 (H11N9)
 A/Mallard (Anas platyrhynchos)/South Korea/KNU2019-30/2019 (H7N7)
 Eurasian AIVs
 Eurasian AIVs
 A/cape shoveller/South Africa/STR0982/2013 (H4N8)
 European AIVs
 A/avian/Egypt/920431/2006 (H9N2)
 A/duck/Mongolia/543/2015 (H4N6)
 A/duck/Vietnam/NCVD-ND4V3P/2016 (H3N8)
 A/duck/Aichi/231003/2016 (H8N4)
 A/environment/Kagoshima/KU-G4/2020 (H1N1)
 A/duck/Jiangxi/SE2073/2017 (H6N1)
88
81
95
99
99
99
96
83
99
99
99
94
99
96
98
99
99
99
88
95
84
99
99
83
74
99
99
99
79
73
99
99
99
97
99
99
 A/duck/Mongolia/419/2019 (H5N3)
 A/whooper swan/Shanxi/SX116/2020 (H5N2)
 A/environment/Kagoshima/KU-3c/2019 (H11N2)
 A/White-fronted goose(Anser albifrons)/South Korea/KNU2019-37/2019 (H1N1)
 A/mallard/Kagoshima/KG1C/2017 (H4N9)
 A/wild waterfowl/Korea/F7-18/2018 (H4N8)
 A/Caspian seal/Russia/T1/2012 (H4N6)
 A/chicken/Wales/600-020823/2014 (H4N6)
 A/mallard/Sweden/4/2005 (H10N4)
 Asian AIVs]
 A/chicken/Iran/SS4/2009 (H9N2)
 A/chicken/Iran/SS7/2011 (H9N2)
 Eurasian AIVs
 Asian AIVs
 A/duck/Hokkaido/Vac-2/2004 (H7N7)
 European AIVs
 European AIVs
 A/mallard/Bavaria/185-8/2008 (H1N1)
 A/duck/Bangladesh/17D1710/2021 (H5N1)
 A/duck/Hokkaido/Vac-3/2007 (H5N1)
 A/avian/Japan/8KI0184/2008 (H4N6)
 A/breeder duck/Korea/Gochang1/2014 (H5N8)
 A/duck/Mongolia/572 (H3N8)
 A/environment/Kagoshima/KU-D3/2019 (H3N8)
 A/duck/Kyoto/260112/2016 (H1N1)
 A/duck/Tokushima/361020/2015 (H6N2)
 Asian AIVs
 A/African starling/England-Q/983/1979 (H7N1)
 A/duck/Shimane/188/1999 (H1N1)
 A/duck/Kingmen/E322/04 (H6N2)
 A/environment/Kagoshima/KU-3a/2019 (H6N6)
 A/environment/Kagoshima/KU-6b/2019 (H6N6)
 A/environment/Kagoshima/KU-4a/2019 (H6N6)
 A/environment/Kagoshima/KU-5a/2019 (H6N6)
 A/environment/Kagoshima/KU-B1/2019 (H6N6)
 A/environment/Kagoshima/KU-3b/2019 (H6N6)
 A/environment/Kagoshima/KU-G1/2019 (H6N6)
 A/Northern Pintail/Russia Primorje/137/2019 (H6N6)
 A/Mallard(Anas platyrhynchos)/South Korea/KNU 2019-49/2019 (H6N6)
 A/mallard/Amur region/81b/2019 (H6N6)
 A/Mallard (Anas platyrhynchos)/South Korea/KNU2019-61/2019 (H4N6)
 A/duck/China/330D17/2018 (H6N6)
 A/duck/Guizhou/8.26 ZYLJJ020-C/2018 (H6N6)
 A/duck/Hunan/1.17 YYGKK97-OC/2017 (H6N6)
 Asian AIVs
 A/finch/England/2051/1991 (H5N2)
 A/black-headed gull/Netherlands/29/2014 (H13N6)
 A/Larus argentatus/Belgium/U1610336 H907447/2016 (H16N3)
 A/seagull/Chile/5775/2009 (H13N9)
 A/Glaucous-Winged Gull/Alaska/19MB02474/2019 (H16N3)
 Eurasian AIVs
 A/duck/Cambodia/b0120501/2017 (H7N3)
 Asian AIVs
 A/chicken/Queensland/1/2012 (H10N7)
 A/duck/Victoria/2001 (H4N6)
 A/slaty-backed gull/Japan/6KS0185/2006 (H4N8)
 Asian AIVs
 Eurasian AIVs
 A/duck/Hong Kong/702/1979 (H9N2)
 A/shearwater/AUS/405/1978 (H3N8)
 A/baikal teal/Korea/Donglim3/2014 (H5N8)
 A/chicken/Kumamoto/1-7/2014 (H5N8)
99
99
81
99
74
98
93
99
90
99
99
99
99
92
79
76
88
98
99
99
92
71
73
99
99
97
99
73
99
93
99
99
97
76
99
75
99
72
88
99
 A/crane/Kagoshima/KU53/2015 (H5N8)
99
 A/duck/Guangdong/wy11/2008 (H5N5)
84
 Asian AIVs
 A/duck/Hokkaido/Vac-1/2004 (H5N1)
 A/duck/Nanchang/4-165/2000 (H4N6)
 A/environment/Kagoshima/KU-4c/2019 (H4N6)
75
 A/environment/Kagoshima/KU-6f/2020 (H4N6)
99
 A/duck/Miyazaki/450110/2016 (H9N2)
 A/teal/Toguchin/1157/2016 (H6N1)
85
 Asian AIVs
91
 A/duck/NZL/41/1984 (H1N2)
 A/red-necked stint/Australia/4189/1980 (H4N8)
90
 Nolrth American AIVs
0.05
PB2
PB2-I
PB2-II
PB2-III
PB2-IV
Fig. S1a

## Slide 2
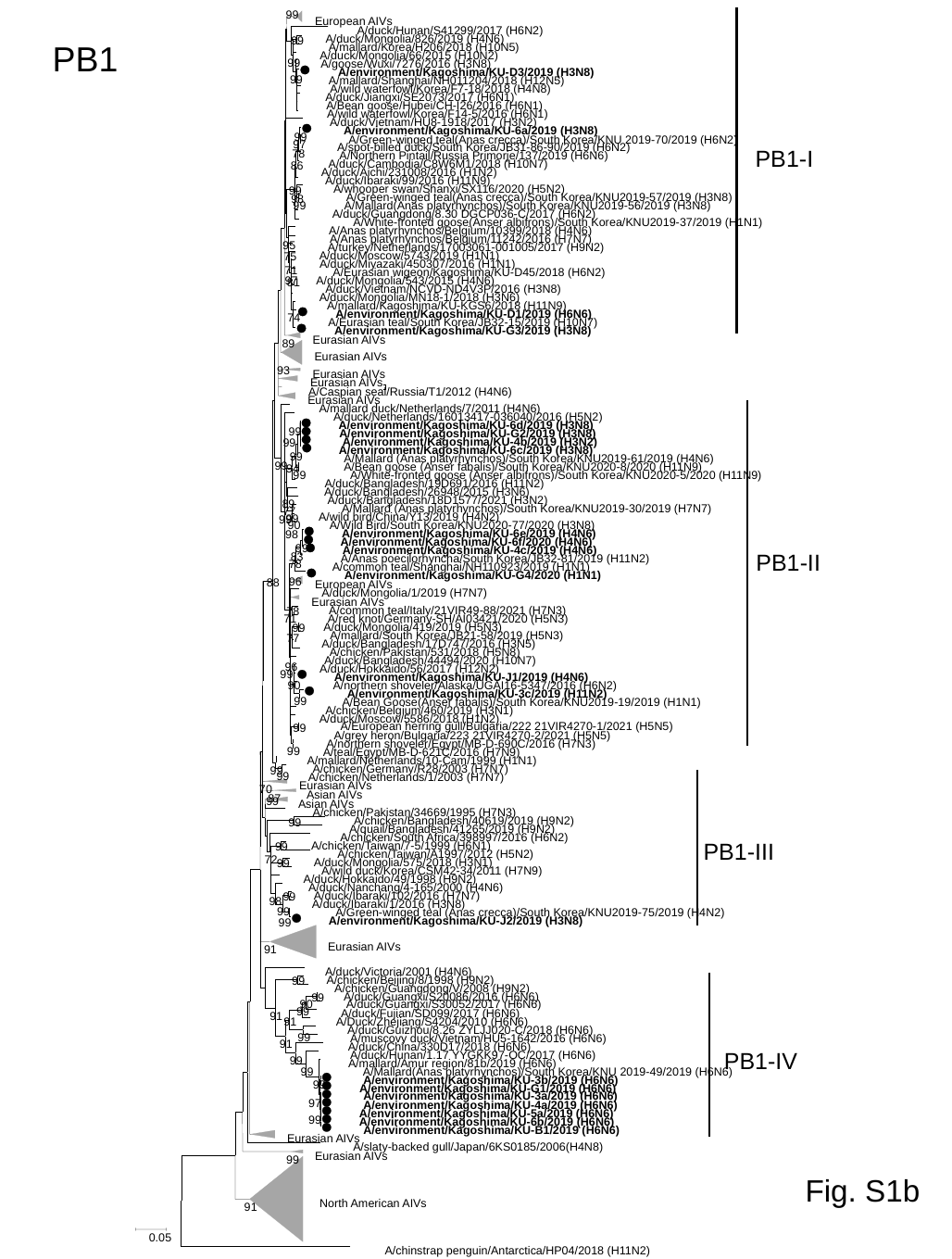

99
 European AIVs
 A/duck/Hunan/S41299/2017 (H6N2)
 A/duck/Mongolia/826/2019 (H4N6)
 A/mallard/Korea/H206/2018 (H10N5)
 A/duck/Mongolia/66/2015 (H10N2)
 A/goose/Wuxi/7276/2016 (H3N8)
 A/environment/Kagoshima/KU-D3/2019 (H3N8)
 A/mallard/Shanghai/NH011204/2018 (H12N5)
 A/wild waterfowl/Korea/F7-18/2018 (H4N8)
 A/duck/Jiangxi/SE2073/2017 (H6N1)
 A/Bean goose/Hubei/CH-I26/2016 (H6N1)
 A/wild waterfowl/Korea/F14-5/2016 (H6N1)
 A/duck/Vietnam/HU8-1918/2017 (H3N2)
 A/environment/Kagoshima/KU-6a/2019 (H3N8)
 A/Green-winged teal(Anas crecca)/South Korea/KNU 2019-70/2019 (H6N2)
 A/spot-billed duck/South Korea/JB31-86-90/2019 (H6N2)
 A/Northern Pintail/Russia Primorje/137/2019 (H6N6)
 A/duck/Cambodia/C8W6M1/2018 (H10N7)
 A/duck/Aichi/231008/2016 (H1N2)
 A/duck/Ibaraki/99/2016 (H11N9)
 A/whooper swan/Shanxi/SX116/2020 (H5N2)
 A/Green-winged teal(Anas crecca)/South Korea/KNU2019-57/2019 (H3N8)
 A/Mallard(Anas platyrhynchos)/South Korea/KNU2019-56/2019 (H3N8)
 A/duck/Guangdong/8.30 DGCP036-C/2017 (H6N2)
 A/White-fronted goose(Anser albifrons)/South Korea/KNU2019-37/2019 (H1N1)
 A/Anas platyrhynchos/Belgium/10399/2018 (H4N6)
 A/Anas platyrhynchos/Belgium/11242/2016 (H7N7)
 A/turkey/Netherlands/17003061-001005/2017 (H9N2)
 A/duck/Moscow/5743/2019 (H1N1)
 A/duck/Miyazaki/450307/2016 (H1N1)
 A/Eurasian wigeon/Kagoshima/KU-D45/2018 (H6N2)
 A/duck/Mongolia/543/2015 (H4N6)
 A/duck/Vietnam/NCVD-ND4V3P/2016 (H3N8)
 A/duck/Mongolia/MN18-1/2018 (H3N6)
 A/mallard/Kagoshima/KU-KGS6/2018 (H11N9)
 A/environment/Kagoshima/KU-D1/2019 (H6N6)
 A/Eurasian teal/South Korea/JB32-15/2019 (H10N7)
 A/environment/Kagoshima/KU-G3/2019 (H3N8)
 Eurasian AIVs
 Eurasian AIVs
 Eurasian AIVs
 Eurasian AIVs
 A/Caspian seal/Russia/T1/2012 (H4N6)
 Eurasian AIVs
 A/mallard duck/Netherlands/7/2011 (H4N6)
 A/duck/Netherlands/16013417-036040/2016 (H5N2)
 A/environment/Kagoshima/KU-6d/2019 (H3N8)
 A/environment/Kagoshima/KU-G2/2019 (H3N8)
 A/environment/Kagoshima/KU-4b/2019 (H3N2)
 A/environment/Kagoshima/KU-6c/2019 (H3N8)
 A/Mallard (Anas platyrhynchos)/South Korea/KNU2019-61/2019 (H4N6)
 A/Bean goose (Anser fabalis)/South Korea/KNU2020-8/2020 (H11N9)
 A/White-fronted goose (Anser albifrons)/South Korea/KNU2020-5/2020 (H11N9)
 A/duck/Bangladesh/19D691/2016 (H11N2)
 A/duck/Bangladesh/26948/2015 (H3N6)
 A/duck/Bangladesh/18D1577/2021 (H3N2)
 A/Mallard (Anas platyrhynchos)/South Korea/KNU2019-30/2019 (H7N7)
 A/wild bird/China/Y13/2019 (H4N2)
 A/Wild Bird/South Korea/KNU2020-77/2020 (H3N8)
 A/environment/Kagoshima/KU-6e/2019 (H4N6)
 A/environment/Kagoshima/KU-6f/2020 (H4N6)
 A/environment/Kagoshima/KU-4c/2019 (H4N6)
 A/Anas poecilorhyncha/South Korea/JB32-81/2019 (H11N2)
 A/common teal/Shanghai/NH110923/2019 (H1N1)
 A/environment/Kagoshima/KU-G4/2020 (H1N1)
 European AIVs
 A/duck/Mongolia/1/2019 (H7N7)
99
99
99
99
97
78
86
99
98
99
95
75
71
97
81
74
89
93
 Eurasian AIVs
 A/common teal/Italy/21VIR49-88/2021 (H7N3)
 A/red knot/Germany-SH/AI03421/2020 (H5N3)
 A/duck/Mongolia/419/2019 (H5N3)
 A/mallard/South Korea/JB21-58/2019 (H5N3)
 A/duck/Bangladesh/17D747/2016 (H3N5)
 A/chicken/Pakistan/531/2018 (H5N8)
 A/duck/Bangladesh/44494/2020 (H10N7)
 A/duck/Hokkaido/56/2017 (H12N2)
 A/environment/Kagoshima/KU-J1/2019 (H4N6)
 A/northern shoveler/Alaska/UGAI16-5347/2016 (H6N2)
 A/environment/Kagoshima/KU-3c/2019 (H11N2)
 A/Bean Goose(Anser fabalis)/South Korea/KNU2019-19/2019 (H1N1)
 A/chicken/Belgium/460/2019 (H3N1)
 A/duck/Moscow/5586/2018 (H1N2)
 A/European herring gull/Bulgaria/222 21VIR4270-1/2021 (H5N5)
 A/grey heron/Bulgaria/223 21VIR4270-2/2021 (H5N5)
 A/northern shoveler/Egypt/MB-D-690C/2016 (H7N3)
 A/teal/Egypt/MB-D-621C/2016 (H7N9)
 A/mallard/Netherlands/10-Cam/1999 (H1N1)
 A/chicken/Germany/R28/2003 (H7N7)
 A/chicken/Netherlands/1/2003 (H7N7)
 Eurasian AIVs
 Asian AIVs
 Asian AIVs
 A/chicken/Pakistan/34669/1995 (H7N3)
 A/chicken/Bangladesh/40619/2019 (H9N2)
 A/quail/Bangladesh/41265/2019 (H9N2)
 A/chicken/South Africa/398997/2016 (H6N2)
 A/chicken/Taiwan/7-5/1999 (H6N1)
 A/chicken/Taiwan/A1997/2012 (H5N2)
 A/duck/Mongolia/575/2018 (H3N1)
 A/wild duck/Korea/CSM42-34/2011 (H7N9)
 A/duck/Hokkaido/49/1998 (H9N2)
 A/duck/Nanchang/4-165/2000 (H4N6)
 A/duck/Ibaraki/102/2016 (H7N7)
 A/duck/Ibaraki/1/2016 (H3N8)
 A/Green-winged teal (Anas crecca)/South Korea/KNU2019-75/2019 (H4N2)
 A/environment/Kagoshima/KU-J2/2019 (H3N8)
 Eurasian AIVs
 A/duck/Victoria/2001 (H4N6)
 A/chicken/Beijing/8/1998 (H9N2)
 A/chicken/Guangdong/V/2008 (H9N2)
 A/duck/Guangxi/S20086/2016 (H6N6)
 A/duck/Guangxi/S30052/2017 (H6N6)
 A/duck/Fujian/SD099/2017 (H6N6)
 A/Duck/Zhejiang/S4204/2010 (H6N6)
 A/duck/Guizhou/8.26 ZYLJJ020-C/2018 (H6N6)
 A/muscovy duck/Vietnam/HU5-1642/2016 (H6N6)
 A/duck/China/330D17/2018 (H6N6)
 A/duck/Hunan/1.17 YYGKK97-OC/2017 (H6N6)
 A/mallard/Amur region/81b/2019 (H6N6)
 A/Mallard(Anas platyrhynchos)/South Korea/KNU 2019-49/2019 (H6N6)
 A/environment/Kagoshima/KU-3b/2019 (H6N6)
 A/environment/Kagoshima/KU-G1/2019 (H6N6)
 A/environment/Kagoshima/KU-3a/2019 (H6N6)
 A/environment/Kagoshima/KU-4a/2019 (H6N6)
 A/environment/Kagoshima/KU-5a/2019 (H6N6)
 A/environment/Kagoshima/KU-6b/2019 (H6N6)
 A/environment/Kagoshima/KU-B1/2019 (H6N6)
 Eurasian AIVs
 A/slaty-backed gull/Japan/6KS0185/2006(H4N8)
 Eurasian AIVs
 North American AIVs
99
99
99
99
94
99
89
77
99
99
90
98
99
83
78
96
88
78
71
99
77
96
99
90
99
99
99
99
99
70
87
99
99
99
72
99
99
98
99
99
91
99
99
90
99
91
91
99
91
99
99
99
97
99
99
91
0.05
 A/chinstrap penguin/Antarctica/HP04/2018 (H11N2)
PB1
PB1-I
PB1-II
PB1-III
PB1-IV
Fig. S1b

## Slide 3
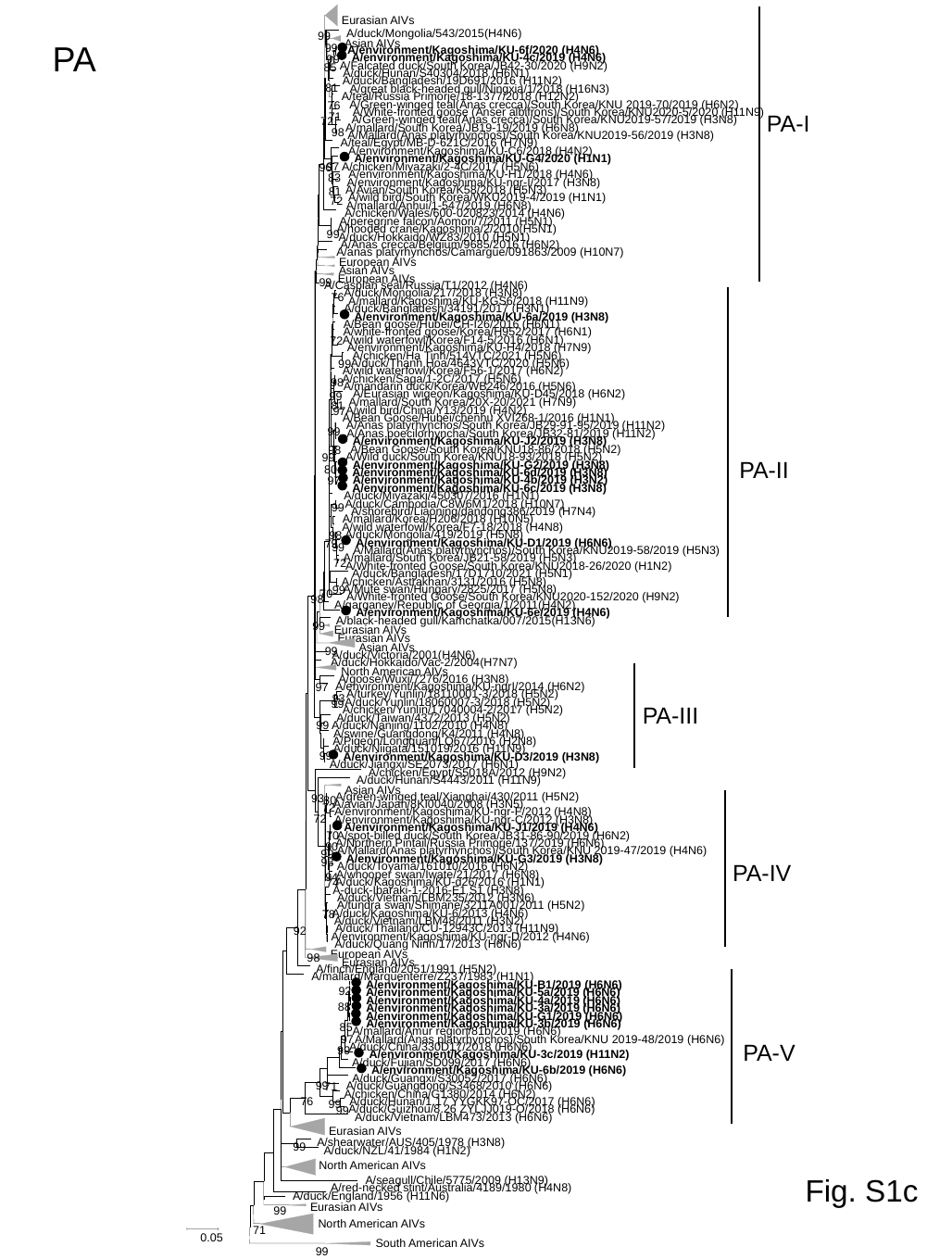

Eurasian AIVs
 A/duck/Mongolia/543/2015(H4N6)
 Asian AIVs
 A/environment/Kagoshima/KU-6f/2020 (H4N6)
 A/environment/Kagoshima/KU-4c/2019 (H4N6)
 A/Falcated duck/South Korea/JB42-30/2020 (H9N2)
 A/duck/Hunan/S40304/2018 (H6N1)
 A/duck/Bangladesh/19D691/2016 (H11N2)
 A/great black-headed gull/Ningxia/1/2018 (H16N3)
 A/teal/Russia Primorje/18-1377/2018 (H12N2)
 A/Green-winged teal(Anas crecca)/South Korea/KNU 2019-70/2019 (H6N2)
 A/White-fronted goose (Anser albifrons)/South Korea/KNU2020-5/2020 (H11N9)
 A/Green-winged teal(Anas crecca)/South Korea/KNU2019-57/2019 (H3N8)
 A/mallard/South Korea/JB19-19/2019 (H6N8)
 A/Mallard(Anas platyrhynchos)/South Korea/KNU2019-56/2019 (H3N8)
 A/teal/Egypt/MB-D-621C/2016 (H7N9)
 A/environment/Kagoshima/KU-C6/2018 (H4N2)
 A/environment/Kagoshima/KU-G4/2020 (H1N1)
 A/chicken/Miyazaki/2-4C/2017 (H5N6)
 A/environment/Kagoshima/KU-H1/2018 (H4N6)
 A/environment/Kagoshima/KU-ngr-I/2017 (H3N8)
 A/Avian/South Korea/K58/2018 (H5N3)
 A/wild bird/South Korea/WKU2019-4/2019 (H1N1)
 A/mallard/Anhui/1-547/2019 (H6N8)
 A/chicken/Wales/600-020823/2014 (H4N6)
 A/peregrine falcon/Aomori/7/2011 (H5N1)
 A/hooded crane/Kagoshima/2/2010(H5N1)
 A/duck/Hokkaido/WZ83/2010 (H5N1)
 A/Anas crecca/Belgium/9685/2016 (H6N2)
 A/anas platyrhynchos/Camargue/091863/2009 (H10N7)
 European AIVs
 Asian AIVs
 European AIVs
 A/Caspian seal/Russia/T1/2012 (H4N6)
 A/duck/Mongolia/217/2018 (H3N8)
 A/mallard/Kagoshima/KU-KGS6/2018 (H11N9)
 A/duck/Bangladesh/34191/2017 (H3N1)
 A/environment/Kagoshima/KU-6a/2019 (H3N8)
 A/Bean goose/Hubei/CH-I26/2016 (H6N1)
 A/white-fronted goose/Korea/H952/2017 (H6N1)
 A/wild waterfowl/Korea/F14-5/2016 (H6N1)
 A/environment/Kagoshima/KU-H4/2018 (H7N9)
 A/chicken/Ha Tinh/514VTC/2021 (H5N6)
 A/duck/Thanh Hoa/4643VTC/2020 (H5N6)
 A/wild waterfowl/Korea/F56-1/2017 (H6N2)
 A/chicken/Saga/1-2C/2017 (H5N6)
 A/mandarin duck/Korea/WB246/2016 (H5N6)
 A/Eurasian wigeon/Kagoshima/KU-D45/2018 (H6N2)
 A/mallard/South Korea/20X-20/2021 (H7N9)
 A/wild bird/China/Y13/2019 (H4N2)
 A/Bean Goose/Hubei/chenhu XVI268-1/2016 (H1N1)
 A/Anas platyrhynchos/South Korea/JB29-91-95/2019 (H11N2)
 A/Anas poecilorhyncha/South Korea/JB32-81/2019 (H11N2)
 A/environment/Kagoshima/KU-J2/2019 (H3N8)
 A/Bean Goose/South Korea/KNU18-86/2018 (H5N2)
 A/Wild duck/South Korea/KNU18-93/2018 (H5N2)
 A/environment/Kagoshima/KU-G2/2019 (H3N8)
 A/environment/Kagoshima/KU-6d/2019 (H3N8)
 A/environment/Kagoshima/KU-4b/2019 (H3N2)
 A/environment/Kagoshima/KU-6c/2019 (H3N8)
 A/duck/Miyazaki/450307/2016 (H1N1)
 A/duck/Cambodia/C8W6M1/2018 (H10N7)
 A/shorebird/Liaoning/dandong386/2019 (H7N4)
 A/mallard/Korea/H206/2018 (H10N5)
 A/wild waterfowl/Korea/F7-18/2018 (H4N8)
 A/duck/Mongolia/419/2019 (H5N8)
 A/environment/Kagoshima/KU-D1/2019 (H6N6)
 A/Mallard(Anas platyrhynchos)/South Korea/KNU2019-58/2019 (H5N3)
99
99
99
85
81
76
71
72
98
97
96
83
81
72
99
 A/mallard/South Korea/JB21-58/2019 (H5N3)
 A/White-fronted Goose/South Korea/KNU2018-26/2020 (H1N2)
 A/duck/Bangladesh/17D1710/2021 (H5N1)
 A/chicken/Astrakhan/3131/2016 (H5N8)
 A/Mute swan/Hungary/2825/2017 (H5N8)
 A/White-fronted Goose/South Korea/KNU2020-152/2020 (H9N2)
 A/garganey/Republic of Georgia/1/2011(H4N2)
 A/environment/Kagoshima/KU-6e/2019 (H4N6)
 A/black-headed gull/Kamchatka/007/2015(H13N6)
 Eurasian AIVs
 Eurasian AIVs
 Asian AIVs
 A/duck/Victoria/2001(H4N6)
 A/duck/Hokkaido/Vac-2/2004(H7N7)
 North American AIVs
 A/goose/Wuxi/7276/2016 (H3N8)
 A/environment/Kagoshima/KU-ngrI/2014 (H6N2)
 A/turkey/Yunlin/18110001-3/2018 (H5N2)
 A/duck/Yunlin/18060007-3/2018 (H5N2)
 A/chicken/Yunlin/17040004-2/2017 (H5N2)
 A/duck/Taiwan/4372/2013 (H5N2)
 A/duck/Nanjing/1102/2010 (H4N8)
 A/swine/Guangdong/K4/2011 (H4N8)
 A/Pigeon/Longquan/LQ67/2016 (H2N8)
 A/duck/Niigata/151019/2016 (H11N9)
 A/environment/Kagoshima/KU-D3/2019 (H3N8)
 A/duck/Jiangxi/SE2073/2017 (H6N1)
 A/chicken/Egypt/S5018A/2012 (H9N2)
 A/duck/Hunan/S4443/2011 (H11N9)
 Asian AIVs
 A/green-winged teal/Xianghai/430/2011 (H5N2)
 A/avian/Japan/8KI0040/2008 (H3N5)
 A/environment/Kagoshima/KU-ngr-F/2012 (H4N8)
 A/environment/Kagoshima/KU-ngr-C/2012 (H3N8)
 A/environment/Kagoshima/KU-J1/2019 (H4N6)
 A/spot-billed duck/South Korea/JB31-86-90/2019 (H6N2)
 A/Northern Pintail/Russia Primorje/137/2019 (H6N6)
 A/Mallard(Anas platyrhynchos)/South Korea/KNU 2019-47/2019 (H4N6)
 A/environment/Kagoshima/KU-G3/2019 (H3N8)
 A/duck/Toyama/161010/2016 (H6N2)
 A/whooper swan/Iwate/21/2017 (H6N8)
 A/duck/Kagoshima/KU-d26/2016 (H1N1)
 A-duck-Ibaraki-1-2016-E1 S1 (H3N8)
 A/duck/Vietnam/LBM235/2012 (H3N6)
 A/tundra swan/Shimane/3211A001/2011 (H5N2)
 A/duck/Kagoshima/KU-6/2013 (H4N6)
 A/duck/Vietnam/LBM48/2011 (H3N2)
 A/duck/Thailand/CU-12943C/2013 (H11N9)
 A/environment/Kagoshima/KU-ngr-D/2012 (H4N6)
 A/duck/Quang Ninh/17/2013 (H6N6)
 European AIVs
 Eurasian AIVs
 A/finch/England/2051/1991 (H5N2)
 A/mallard/Marquenterre/Z237/1983 (H1N1)
 A/environment/Kagoshima/KU-B1/2019 (H6N6)
 A/environment/Kagoshima/KU-5a/2019 (H6N6)
 A/environment/Kagoshima/KU-4a/2019 (H6N6)
 A/environment/Kagoshima/KU-3a/2019 (H6N6)
 A/environment/Kagoshima/KU-G1/2019 (H6N6)
 A/environment/Kagoshima/KU-3b/2019 (H6N6)
 A/mallard/Amur region/81b/2019 (H6N6)
 A/Mallard(Anas platyrhynchos)/South Korea/KNU 2019-48/2019 (H6N6)
99
76
72
99
98
99
81
97
99
98
99
80
97
99
98
79
99
72
99
70
98
99
99
97
93
99
99
99
93
80
72
72
70
90
99
98
94
74
78
92
98
92
88
85
97
 A/duck/China/330D17/2018 (H6N6)
99
 A/environment/Kagoshima/KU-3c/2019 (H11N2)
 A/duck/Fujian/SD099/2017 (H6N6)
 A/environment/Kagoshima/KU-6b/2019 (H6N6)
 A/duck/Guangxi/S30052/2017 (H6N6)
99
 A/duck/Guangdong/S3468/2010 (H6N6)
71
 A/chicken/China/G1380/2014 (H6N2)
76
 A/duck/Hunan/1.17 YYGKK97-OC/2017 (H6N6)
99
 A/duck/Guizhou/8.26 ZYLJJ019-O/2018 (H6N6)
99
 A/duck/Vietnam/LBM473/2013 (H6N6)
 Eurasian AIVs
 A/shearwater/AUS/405/1978 (H3N8)
99
 A/duck/NZL/41/1984 (H1N2)
 North American AIVs
 A/seagull/Chile/5775/2009 (H13N9)
 A/red-necked stint/Australia/4189/1980 (H4N8)
 A/duck/England/1956 (H11N6)
 Eurasian AIVs
99
 North American AIVs
71
0.05
 South American AIVs
99
PA
PA-I
PA-II
PA-III
PA-IV
PA-V
Fig. S1c

## Slide 4
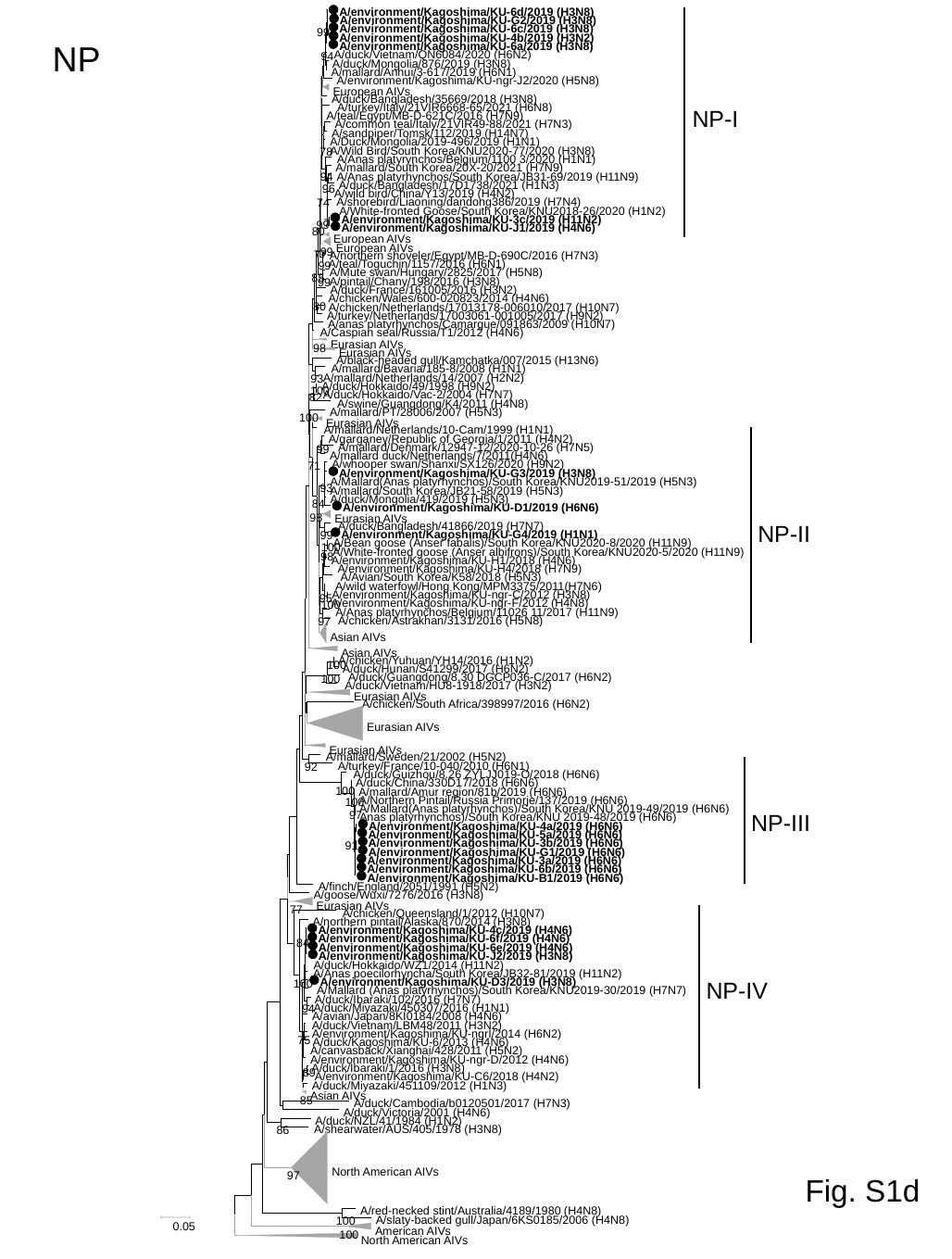

A/environment/Kagoshima/KU-6d/2019 (H3N8)
 A/environment/Kagoshima/KU-G2/2019 (H3N8)
 A/environment/Kagoshima/KU-6c/2019 (H3N8)
 A/environment/Kagoshima/KU-4b/2019 (H3N2)
 A/environment/Kagoshima/KU-6a/2019 (H3N8)
 A/duck/Vietnam/QN6084/2020 (H6N2)
 A/duck/Mongolia/876/2019 (H3N8)
 A/mallard/Anhui/3-617/2019 (H6N1)
 A/environment/Kagoshima/KU-ngr-J2/2020 (H5N8)
 European AIVs
 A/duck/Bangladesh/35669/2018 (H3N8)
 A/turkey/Italy/21VIR6668-65/2021 (H6N8)
 A/teal/Egypt/MB-D-621C/2016 (H7N9)
 A/common teal/Italy/21VIR49-88/2021 (H7N3)
 A/sandpiper/Tomsk/112/2019 (H14N7)
 A/Duck/Mongolia/2019-496/2019 (H1N1)
 A/Wild Bird/South Korea/KNU2020-77/2020 (H3N8)
 A/Anas platyrynchos/Belgium/1100 3/2020 (H1N1)
 A/mallard/South Korea/20X-20/2021 (H7N9)
 A/Anas platyrhynchos/South Korea/JB31-69/2019 (H11N9)
 A/duck/Bangladesh/17D1738/2021 (H1N3)
 A/wild bird/China/Y13/2019 (H4N2)
 A/shorebird/Liaoning/dandong386/2019 (H7N4)
 A/White-fronted Goose/South Korea/KNU2018-26/2020 (H1N2)
 A/environment/Kagoshima/KU-3c/2019 (H11N2)
 A/environment/Kagoshima/KU-J1/2019 (H4N6)
 European AIVs
 European AIVs
 A/northern shoveler/Egypt/MB-D-690C/2016 (H7N3)
 A/teal/Toguchin/1157/2016 (H6N1)
 A/Mute swan/Hungary/2825/2017 (H5N8)
 A/pintail/Chany/198/2016 (H3N8)
 A/duck/France/161005/2016 (H3N2)
 A/chicken/Wales/600-020823/2014 (H4N6)
 A/chicken/Netherlands/17013178-006010/2017 (H10N7)
 A/turkey/Netherlands/17003061-001005/2017 (H9N2)
 A/anas platyrhynchos/Camargue/091863/2009 (H10N7)
 A/Caspian seal/Russia/T1/2012 (H4N6)
 Eurasian AIVs
 Eurasian AIVs
 A/black-headed gull/Kamchatka/007/2015 (H13N6)
 A/mallard/Bavaria/185-8/2008 (H1N1)
 A/mallard/Netherlands/14/2007 (H2N2)
 A/duck/Hokkaido/49/1998 (H9N2)
 A/duck/Hokkaido/Vac-2/2004 (H7N7)
 A/swine/Guangdong/K4/2011 (H4N8)
 A/mallard/PT/28006/2007 (H5N3)
 Eurasian AIVs
 A/mallard/Netherlands/10-Cam/1999 (H1N1)
 A/garganey/Republic of Georgia/1/2011 (H4N2)
 A/mallard/Denmark/12947-12/2020-10-26 (H7N5)
 A/mallard duck/Netherlands/7/2011(H4N6)
 A/whooper swan/Shanxi/SX126/2020 (H9N2)
 A/environment/Kagoshima/KU-G3/2019 (H3N8)
 A/Mallard(Anas platyrhynchos)/South Korea/KNU2019-51/2019 (H5N3)
 A/mallard/South Korea/JB21-58/2019 (H5N3)
 A/duck/Mongolia/419/2019 (H5N3)
 A/environment/Kagoshima/KU-D1/2019 (H6N6)
 Eurasian AIVs
 A/duck/Bangladesh/41866/2019 (H7N7)
 A/environment/Kagoshima/KU-G4/2019 (H1N1)
 A/Bean goose (Anser fabalis)/South Korea/KNU2020-8/2020 (H11N9)
 A/White-fronted goose (Anser albifrons)/South Korea/KNU2020-5/2020 (H11N9)
 A/environment/Kagoshima/KU-H1/2018 (H4N6)
 A/environment/Kagoshima/KU-H4/2018 (H7N9)
 A/Avian/South Korea/K58/2018 (H5N3)
 A/wild waterfowl/Hong Kong/MPM3375/2011(H7N6)
 A/environment/Kagoshima/KU-ngr-C/2012 (H3N8)
99
94
78
94
96
74
77
99
80
99
77
99
85
99
80
 A/environment/Kagoshima/KU-ngr-F/2012 (H4N8)
 A/Anas platyrhynchos/Belgium/11026 11/2017 (H11N9)
 A/chicken/Astrakhan/3131/2016 (H5N8)
 Asian AIVs
 Asian AIVs
 A/chicken/Yuhuan/YH14/2016 (H1N2)
 A/duck/Hunan/S41299/2017 (H6N2)
 A/duck/Guangdong/8.30 DGCP036-C/2017 (H6N2)
 A/duck/Vietnam/HU8-1918/2017 (H3N2)
 Eurasian AIVs
 A/chicken/South Africa/398997/2016 (H6N2)
 Eurasian AIVs
 Eurasian AIVs
 A/mallard/Sweden/21/2002 (H5N2)
 A/turkey/France/10-040/2010 (H6N1)
 A/duck/Guizhou/8.26 ZYLJJ019-O/2018 (H6N6)
 A/duck/China/330D17/2018 (H6N6)
 A/mallard/Amur region/81b/2019 (H6N6)
 A/Northern Pintail/Russia Primorje/137/2019 (H6N6)
 A/Mallard(Anas platyrhynchos)/South Korea/KNU 2019-49/2019 (H6N6)
 Anas platyrhynchos)/South Korea/KNU 2019-48/2019 (H6N6)
 A/environment/Kagoshima/KU-4a/2019 (H6N6)
 A/environment/Kagoshima/KU-5a/2019 (H6N6)
 A/environment/Kagoshima/KU-3b/2019 (H6N6)
 A/environment/Kagoshima/KU-G1/2019 (H6N6)
 A/environment/Kagoshima/KU-3a/2019 (H6N6)
 A/environment/Kagoshima/KU-6b/2019 (H6N6)
 A/environment/Kagoshima/KU-B1/2019 (H6N6)
 A/finch/England/2051/1991 (H5N2)
 A/goose/Wuxi/7276/2016 (H3N8)
 Eurasian AIVs
 A/chicken/Queensland/1/2012 (H10N7)
 A/northern pintail/Alaska/870/2014 (H3N8)
 A/environment/Kagoshima/KU-4c/2019 (H4N6)
 A/environment/Kagoshima/KU-6f/2019 (H4N6)
 A/environment/Kagoshima/KU-6e/2019 (H4N6)
 A/environment/Kagoshima/KU-J2/2019 (H3N8)
 A/duck/Hokkaido/WZ1/2014 (H11N2)
 A/Anas poecilorhyncha/South Korea/JB32-81/2019 (H11N2)
 A/environment/Kagoshima/KU-D3/2019 (H3N8)
 A/Mallard (Anas platyrhynchos)/South Korea/KNU2019-30/2019 (H7N7)
 A/duck/Ibaraki/102/2016 (H7N7)
 A/duck/Miyazaki/450307/2016 (H1N1)
 A/avian/Japan/8KI0184/2008 (H4N6)
 A/duck/Vietnam/LBM48/2011 (H3N2)
 A/environment/Kagoshima/KU-ngrI/2014 (H6N2)
 A/duck/Kagoshima/KU-6/2013 (H4N6)
 A/canvasback/Xianghai/428/2011 (H5N2)
 A/environment/Kagoshima/KU-ngr-D/2012 (H4N6)
 A/duck/Ibaraki/1/2016 (H3N8)
 A/environment/Kagoshima/KU-C6/2018 (H4N2)
 A/duck/Miyazaki/451109/2012 (H1N3)
 Asian AIVs
 A/duck/Cambodia/b0120501/2017 (H7N3)
 A/duck/Victoria/2001 (H4N6)
 A/duck/NZL/41/1984 (H1N2)
 A/shearwater/AUS/405/1978 (H3N8)
 North American AIVs
 A/red-necked stint/Australia/4189/1980 (H4N8)
 A/slaty-backed gull/Japan/6KS0185/2006 (H4N8)
 American AIVs
98
93
100
82
100
99
71
93
84
98
99
100
98
95
100
97
100
100
92
100
100
97
91
77
84
100
94
75
89
85
86
97
100
0.05
100
 North American AIVs
NP
NP-I
NP-II
NP-III
NP-IV
Fig. S1d

## Slide 5
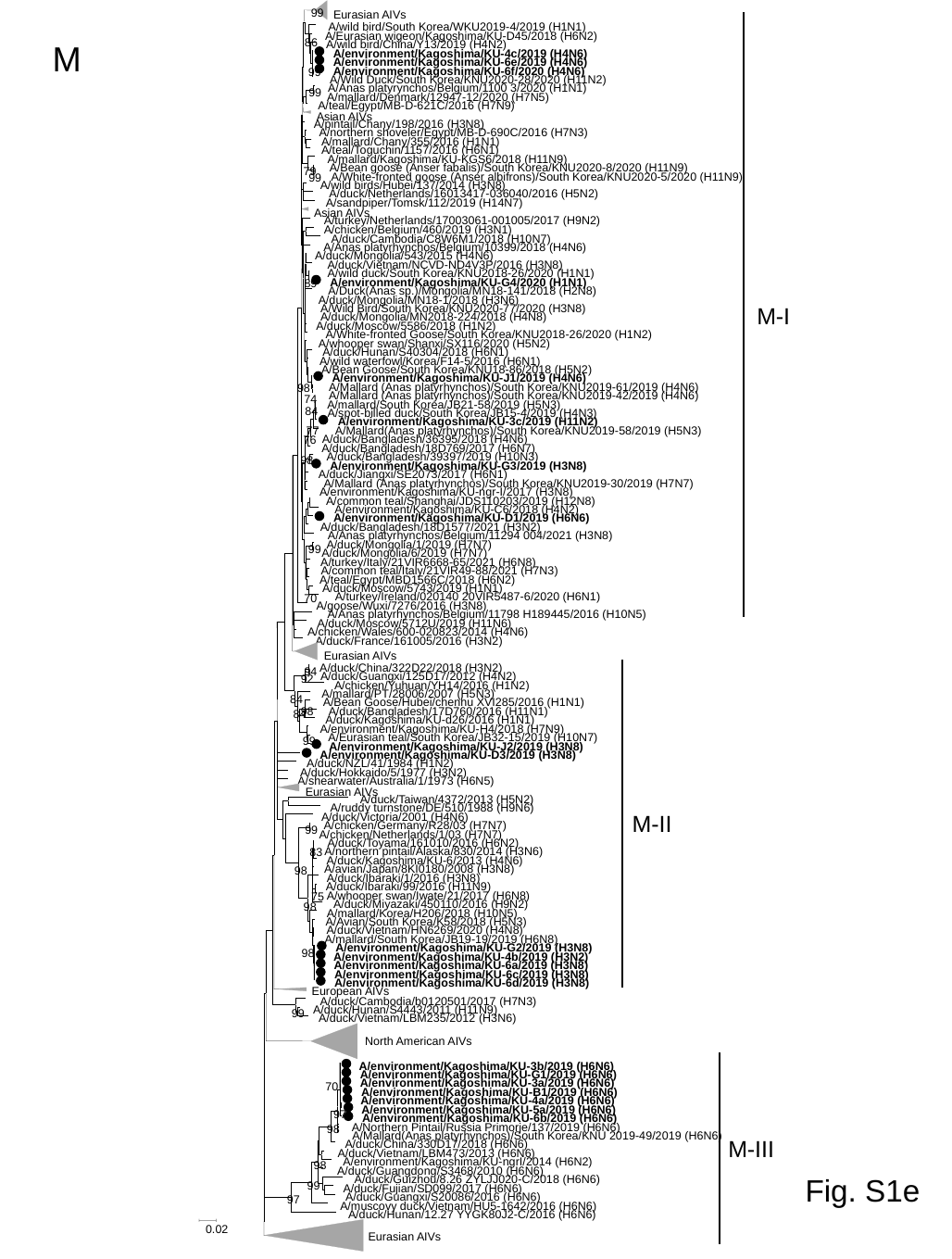

Eurasian AIVs
 A/wild bird/South Korea/WKU2019-4/2019 (H1N1)
 A/Eurasian wigeon/Kagoshima/KU-D45/2018 (H6N2)
 A/wild bird/China/Y13/2019 (H4N2)
 A/environment/Kagoshima/KU-4c/2019 (H4N6)
 A/environment/Kagoshima/KU-6e/2019 (H4N6)
 A/environment/Kagoshima/KU-6f/2020 (H4N6)
 A/Wild Duck/South Korea/KNU2020-28/2020 (H11N2)
 A/Anas platyrynchos/Belgium/1100 3/2020 (H1N1)
 A/mallard/Denmark/12947-12/2020 (H7N5)
 A/teal/Egypt/MB-D-621C/2016 (H7N9)
 Asian AIVs
 A/pintail/Chany/198/2016 (H3N8)
 A/northern shoveler/Egypt/MB-D-690C/2016 (H7N3)
 A/mallard/Chany/355/2016 (H1N1)
 A/teal/Toguchin/1157/2016 (H6N1)
 A/mallard/Kagoshima/KU-KGS6/2018 (H11N9)
 A/Bean goose (Anser fabalis)/South Korea/KNU2020-8/2020 (H11N9)
 A/White-fronted goose (Anser albifrons)/South Korea/KNU2020-5/2020 (H11N9)
 A/wild birds/Hubei/137/2014 (H3N8)
 A/duck/Netherlands/16013417-036040/2016 (H5N2)
 A/sandpiper/Tomsk/112/2019 (H14N7)
 Asian AIVs
 A/turkey/Netherlands/17003061-001005/2017 (H9N2)
 A/chicken/Belgium/460/2019 (H3N1)
 A/duck/Cambodia/C8W6M1/2018 (H10N7)
 A/Anas platyrhynchos/Belgium/10399/2018 (H4N6)
 A/duck/Mongolia/543/2015 (H4N6)
 A/duck/Vietnam/NCVD-ND4V3P/2016 (H3N8)
 A/wild duck/South Korea/KNU2018-26/2020 (H1N1)
 A/environment/Kagoshima/KU-G4/2020 (H1N1)
 A/Duck(Anas sp.)/Mongolia/MN18-141/2018 (H2N8)
 A/duck/Mongolia/MN18-1/2018 (H3N6)
 A/Wild Bird/South Korea/KNU2020-77/2020 (H3N8)
 A/duck/Mongolia/MN2018-224/2018 (H4N8)
 A/duck/Moscow/5586/2018 (H1N2)
 A/White-fronted Goose/South Korea/KNU2018-26/2020 (H1N2)
 A/whooper swan/Shanxi/SX116/2020 (H5N2)
 A/duck/Hunan/S40304/2018 (H6N1)
 A/wild waterfowl/Korea/F14-5/2016 (H6N1)
 A/Bean Goose/South Korea/KNU18-86/2018 (H5N2)
 A/environment/Kagoshima/KU-J1/2019 (H4N6)
 A/Mallard (Anas platyrhynchos)/South Korea/KNU2019-61/2019 (H4N6)
 A/Mallard (Anas platyrhynchos)/South Korea/KNU2019-42/2019 (H4N6)
 A/mallard/South Korea/JB21-58/2019 (H5N3)
 A/spot-billed duck/South Korea/JB15-4/2019 (H4N3)
 A/environment/Kagoshima/KU-3c/2019 (H11N2)
 A/Mallard(Anas platyrhynchos)/South Korea/KNU2019-58/2019 (H5N3)
 A/duck/Bangladesh/36395/2018 (H4N6)
 A/duck/Bangladesh/18D769/2017 (H6N7)
 A/duck/Bangladesh/39397/2019 (H10N3)
 A/environment/Kagoshima/KU-G3/2019 (H3N8)
 A/duck/Jiangxi/SE2073/2017 (H6N1)
 A/Mallard (Anas platyrhynchos)/South Korea/KNU2019-30/2019 (H7N7)
 A/environment/Kagoshima/KU-ngr-I/2017 (H3N8)
 A/common teal/Shanghai/JDS110203/2019 (H12N8)
 A/environment/Kagoshima/KU-C6/2018 (H4N2)
 A/environment/Kagoshima/KU-D1/2019 (H6N6)
 A/duck/Bangladesh/18D1577/2021 (H3N2)
 A/Anas platyrhynchos/Belgium/11294 004/2021 (H3N8)
 A/duck/Mongolia/1/2019 (H7N7)
 A/duck/Mongolia/6/2019 (H7N7)
 A/turkey/Italy/21VIR6668-65/2021 (H6N8)
 A/common teal/Italy/21VIR49-88/2021 (H7N3)
 A/teal/Egypt/MBD1566C/2018 (H6N2)
 A/duck/Moscow/5743/2019 (H1N1)
 A/turkey/Ireland/020140 20VIR5487-6/2020 (H6N1)
 A/goose/Wuxi/7276/2016 (H3N8)
 A/Anas platyrhynchos/Belgium/11798 H189445/2016 (H10N5)
 A/duck/Moscow/5712U/2019 (H11N6)
 A/chicken/Wales/600-020823/2014 (H4N6)
99
86
99
99
79
99
89
98
74
84
77
76
98
99
70
 A/duck/France/161005/2016 (H3N2)
 Eurasian AIVs
 A/duck/China/322D22/2018 (H3N2)
94
 A/duck/Guangxi/125D17/2012 (H4N2)
92
 A/chicken/Yuhuan/YH14/2016 (H1N2)
 A/mallard/PT/28006/2007 (H5N3)
84
 A/Bean Goose/Hubei/chenhu XVI285/2016 (H1N1)
 A/duck/Bangladesh/17D760/2016 (H11N1)
98
84
 A/duck/Kagoshima/KU-d26/2016 (H1N1)
 A/environment/Kagoshima/KU-H4/2018 (H7N9)
 A/Eurasian teal/South Korea/JB32-15/2019 (H10N7)
99
 A/environment/Kagoshima/KU-J2/2019 (H3N8)
 A/environment/Kagoshima/KU-D3/2019 (H3N8)
 A/duck/NZL/41/1984 (H1N2)
 A/duck/Hokkaido/5/1977 (H3N2)
 A/shearwater/Australia/1/1973 (H6N5)
 Eurasian AIVs
 A/duck/Taiwan/4372/2013 (H5N2)
 A/ruddy turnstone/DE/510/1988 (H9N6)
 A/duck/Victoria/2001 (H4N6)
 A/chicken/Germany/R28/03 (H7N7)
99
 A/chicken/Netherlands/1/03 (H7N7)
 A/duck/Toyama/161010/2016 (H6N2)
 A/northern pintail/Alaska/830/2014 (H3N6)
83
 A/duck/Kagoshima/KU-6/2013 (H4N6)
 A/avian/Japan/8KI0180/2008 (H3N8)
98
 A/duck/Ibaraki/1/2016 (H3N8)
 A/duck/Ibaraki/99/2016 (H11N9)
 A/whooper swan/Iwate/21/2017 (H6N8)
75
 A/duck/Miyazaki/450110/2016 (H9N2)
98
 A/mallard/Korea/H206/2018 (H10N5)
 A/Avian/South Korea/K58/2018 (H5N3)
 A/duck/Vietnam/HN6269/2020 (H4N8)
 A/mallard/South Korea/JB19-19/2019 (H6N8)
 A/environment/Kagoshima/KU-G2/2019 (H3N8)
98
 A/environment/Kagoshima/KU-4b/2019 (H3N2)
 A/environment/Kagoshima/KU-6a/2019 (H3N8)
 A/environment/Kagoshima/KU-6c/2019 (H3N8)
 A/environment/Kagoshima/KU-6d/2019 (H3N8)
 European AIVs
 A/duck/Cambodia/b0120501/2017 (H7N3)
 A/duck/Hunan/S4443/2011 (H11N9)
99
 A/duck/Vietnam/LBM235/2012 (H3N6)
 North American AIVs
 A/environment/Kagoshima/KU-3b/2019 (H6N6)
 A/environment/Kagoshima/KU-G1/2019 (H6N6)
 A/environment/Kagoshima/KU-3a/2019 (H6N6)
70
 A/environment/Kagoshima/KU-B1/2019 (H6N6)
 A/environment/Kagoshima/KU-4a/2019 (H6N6)
 A/environment/Kagoshima/KU-5a/2019 (H6N6)
97
 A/environment/Kagoshima/KU-6b/2019 (H6N6)
 A/Northern Pintail/Russia Primorje/137/2019 (H6N6)
98
 A/Mallard(Anas platyrhynchos)/South Korea/KNU 2019-49/2019 (H6N6)
 A/duck/China/330D17/2018 (H6N6)
 A/duck/Vietnam/LBM473/2013 (H6N6)
 A/environment/Kagoshima/KU-ngrI/2014 (H6N2)
98
 A/duck/Guangdong/S3468/2010 (H6N6)
 A/duck/Guizhou/8.26 ZYLJJ020-C/2018 (H6N6)
99
 A/duck/Fujian/SD099/2017 (H6N6)
 A/duck/Guangxi/S20086/2016 (H6N6)
97
 A/muscovy duck/Vietnam/HU5-1642/2016 (H6N6)
 A/duck/Hunan/12.27 YYGK80J2-C/2016 (H6N6)
0.02
 Eurasian AIVs
M
M-I
M-II
M-III
Fig. S1e

## Slide 6
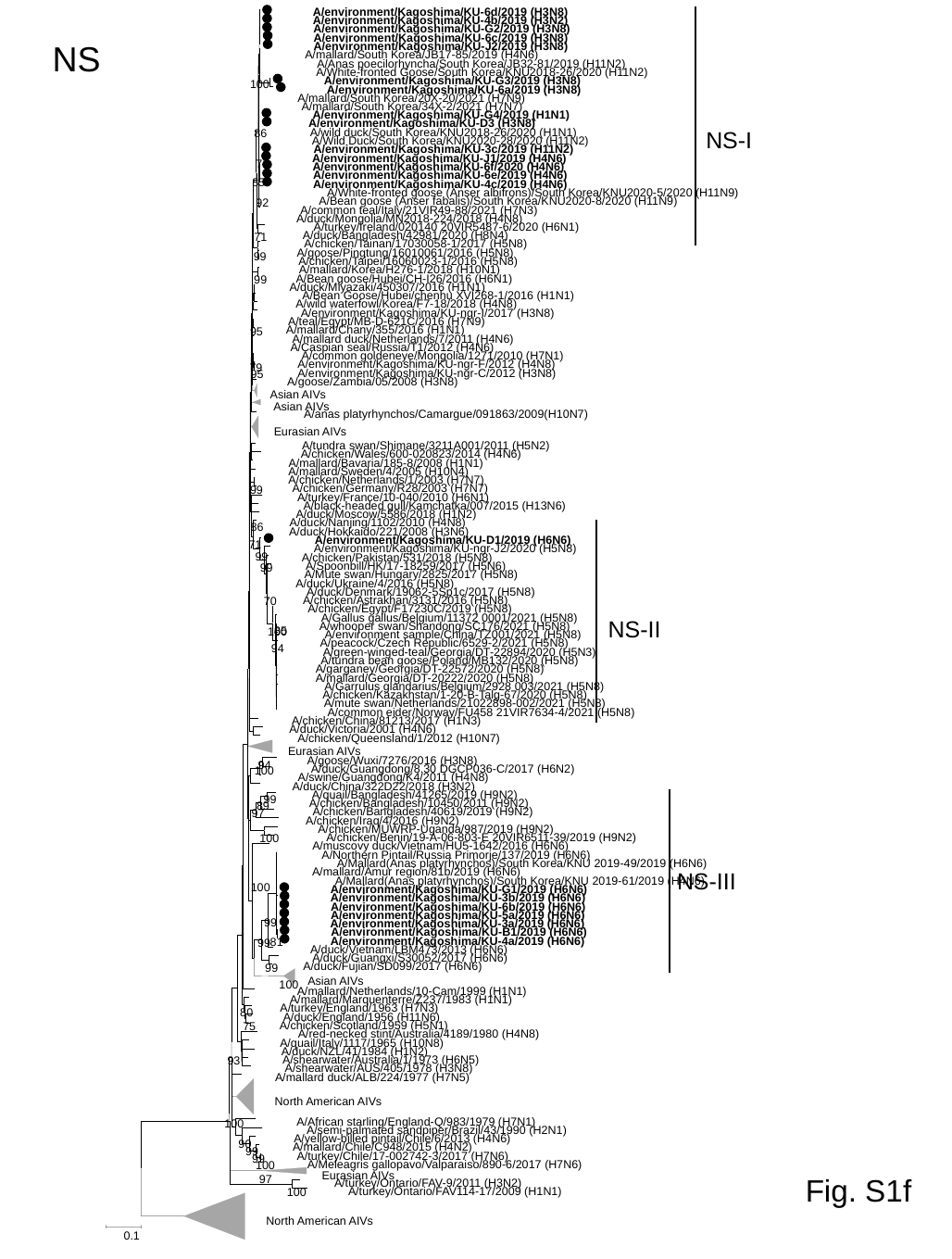

A/environment/Kagoshima/KU-6d/2019 (H3N8)
 A/environment/Kagoshima/KU-4b/2019 (H3N2)
 A/environment/Kagoshima/KU-G2/2019 (H3N8)
 A/environment/Kagoshima/KU-6c/2019 (H3N8)
 A/environment/Kagoshima/KU-J2/2019 (H3N8)
 A/mallard/South Korea/JB17-85/2019 (H4N6)
 A/Anas poecilorhyncha/South Korea/JB32-81/2019 (H11N2)
 A/White-fronted Goose/South Korea/KNU2018-26/2020 (H11N2)
 A/environment/Kagoshima/KU-G3/2019 (H3N8)
 A/environment/Kagoshima/KU-6a/2019 (H3N8)
 A/mallard/South Korea/20X-20/2021 (H7N9)
 A/mallard/South Korea/34X-2/2021 (H7N7)
 A/environment/Kagoshima/KU-G4/2019 (H1N1)
 A/environment/Kagoshima/KU-D3 (H3N8)
 A/wild duck/South Korea/KNU2018-26/2020 (H1N1)
 A/Wild Duck/South Korea/KNU2020-28/2020 (H11N2)
 A/environment/Kagoshima/KU-3c/2019 (H11N2)
 A/environment/Kagoshima/KU-J1/2019 (H4N6)
 A/environment/Kagoshima/KU-6f/2020 (H4N6)
 A/environment/Kagoshima/KU-6e/2019 (H4N6)
 A/environment/Kagoshima/KU-4c/2019 (H4N6)
 A/White-fronted goose (Anser albifrons)/South Korea/KNU2020-5/2020 (H11N9)
 A/Bean goose (Anser fabalis)/South Korea/KNU2020-8/2020 (H11N9)
 A/common teal/Italy/21VIR49-88/2021 (H7N3)
 A/duck/Mongolia/MN2018-224/2018 (H4N8)
 A/turkey/Ireland/020140 20VIR5487-6/2020 (H6N1)
 A/duck/Bangladesh/42981/2020 (H8N4)
 A/chicken/Tainan/17030058-1/2017 (H5N8)
 A/goose/Pingtung/16010061/2016 (H5N8)
 A/chicken/Taipei/16060023-1/2016 (H5N8)
 A/mallard/Korea/H276-1/2018 (H10N1)
 A/Bean goose/Hubei/CH-I26/2016 (H6N1)
 A/duck/Miyazaki/450307/2016 (H1N1)
 A/Bean Goose/Hubei/chenhu XVI268-1/2016 (H1N1)
 A/wild waterfowl/Korea/F7-18/2018 (H4N8)
 A/environment/Kagoshima/KU-ngr-I/2017 (H3N8)
 A/teal/Egypt/MB-D-621C/2016 (H7N9)
 A/mallard/Chany/355/2016 (H1N1)
 A/mallard duck/Netherlands/7/2011 (H4N6)
 A/Caspian seal/Russia/T1/2012 (H4N6)
 A/common goldeneye/Mongolia/1271/2010 (H7N1)
 A/environment/Kagoshima/KU-ngr-F/2012 (H4N8)
 A/environment/Kagoshima/KU-ngr-C/2012 (H3N8)
 A/goose/Zambia/05/2008 (H3N8)
 Asian AIVs
 Asian AIVs
 A/anas platyrhynchos/Camargue/091863/2009(H10N7)
 Eurasian AIVs
 A/tundra swan/Shimane/3211A001/2011 (H5N2)
 A/chicken/Wales/600-020823/2014 (H4N6)
 A/mallard/Bavaria/185-8/2008 (H1N1)
 A/mallard/Sweden/4/2005 (H10N4)
 A/chicken/Netherlands/1/2003 (H7N7)
 A/chicken/Germany/R28/2003 (H7N7)
 A/turkey/France/10-040/2010 (H6N1)
 A/black-headed gull/Kamchatka/007/2015 (H13N6)
 A/duck/Moscow/5586/2018 (H1N2)
 A/duck/Nanjing/1102/2010 (H4N8)
 A/duck/Hokkaido/221/2008 (H3N6)
 A/environment/Kagoshima/KU-D1/2019 (H6N6)
 A/environment/Kagoshima/KU-ngr-J2/2020 (H5N8)
 A/chicken/Pakistan/531/2018 (H5N8)
 A/Spoonbill/HK/17-18259/2017 (H5N6)
 A/Mute swan/Hungary/2825/2017 (H5N8)
 A/duck/Ukraine/4/2016 (H5N8)
 A/duck/Denmark/19062-5Sp1c/2017 (H5N8)
 A/chicken/Astrakhan/3131/2016 (H5N8)
 A/chicken/Egypt/F17230C/2019 (H5N8)
 A/Gallus gallus/Belgium/11372 0001/2021 (H5N8)
 A/whooper swan/Shandong/SC176/2021 (H5N8)
 A/environment sample/China/TZ001/2021 (H5N8)
 A/peacock/Czech Republic/6529-2/2021 (H5N8)
 A/green-winged-teal/Georgia/DT-22894/2020 (H5N3)
 A/tundra bean goose/Poland/MB132/2020 (H5N8)
 A/garganey/Georgia/DT-22572/2020 (H5N8)
 A/mallard/Georgia/DT-20222/2020 (H5N8)
 A/Garrulus glandarius/Belgium/2928 003/2021 (H5N8)
 A/chicken/Kazakhstan/1-20-B-Talg-67/2020 (H5N8)
 A/mute swan/Netherlands/21022898-002/2021 (H5N8)
 A/common eider/Norway/FU458 21VIR7634-4/2021 (H5N8)
 A/chicken/China/81213/2017 (H1N3)
 A/duck/Victoria/2001 (H4N6)
 A/chicken/Queensland/1/2012 (H10N7)
 Eurasian AIVs
 A/goose/Wuxi/7276/2016 (H3N8)
 A/duck/Guangdong/8.30 DGCP036-C/2017 (H6N2)
 A/swine/Guangdong/K4/2011 (H4N8)
 A/duck/China/322D22/2018 (H3N2)
 A/quail/Bangladesh/41265/2019 (H9N2)
 A/chicken/Bangladesh/10450/2011 (H9N2)
 A/chicken/Bangladesh/40619/2019 (H9N2)
 A/chicken/Iraq/4/2016 (H9N2)
 A/chicken/MUWRP-Uganda/987/2019 (H9N2)
 A/chicken/Benin/19-A-06-803-E 20VIR6511-39/2019 (H9N2)
 A/muscovy duck/Vietnam/HU5-1642/2016 (H6N6)
 A/Northern Pintail/Russia Primorje/137/2019 (H6N6)
 A/Mallard(Anas platyrhynchos)/South Korea/KNU 2019-49/2019 (H6N6)
 A/mallard/Amur region/81b/2019 (H6N6)
 A/Mallard(Anas platyrhynchos)/South Korea/KNU 2019-61/2019 (H4N6)
 A/environment/Kagoshima/KU-G1/2019 (H6N6)
 A/environment/Kagoshima/KU-3b/2019 (H6N6)
 A/environment/Kagoshima/KU-6b/2019 (H6N6)
 A/environment/Kagoshima/KU-5a/2019 (H6N6)
 A/environment/Kagoshima/KU-3a/2019 (H6N6)
 A/environment/Kagoshima/KU-B1/2019 (H6N6)
 A/environment/Kagoshima/KU-4a/2019 (H6N6)
 A/duck/Vietnam/LBM473/2013 (H6N6)
 A/duck/Guangxi/S30052/2017 (H6N6)
 A/duck/Fujian/SD099/2017 (H6N6)
 Asian AIVs
 A/mallard/Netherlands/10-Cam/1999 (H1N1)
 A/mallard/Marquenterre/Z237/1983 (H1N1)
 A/turkey/England/1963 (H7N3)
 A/duck/England/1956 (H11N6)
 A/chicken/Scotland/1959 (H5N1)
 A/red-necked stint/Australia/4189/1980 (H4N8)
 A/quail/Italy/1117/1965 (H10N8)
 A/duck/NZL/41/1984 (H1N2)
 A/shearwater/Australia/1/1973 (H6N5)
 A/shearwater/AUS/405/1978 (H3N8)
 A/mallard duck/ALB/224/1977 (H7N5)
 North American AIVs
 A/African starling/England-Q/983/1979 (H7N1)
 A/semi-palmated sandpiper/Brazil/43/1990 (H2N1)
 A/yellow-billed pintail/Chile/6/2013 (H4N6)
 A/mallard/Chile/C948/2015 (H4N2)
 A/turkey/Chile/17-002742-3/2017 (H7N6)
 A/Meleagris gallopavo/Valparaiso/890-6/2017 (H7N6)
 Eurasian AIVs
 A/turkey/Ontario/FAV-9/2011 (H3N2)
 A/turkey/Ontario/FAV114-17/2009 (H1N1)
 North American AIVs
100
86
77
85
92
71
99
99
95
79
95
99
86
71
99
99
70
85
100
94
94
100
99
89
97
100
100
99
81
99
99
100
80
75
93
100
90
99
99
100
97
100
0.1
NS
NS-I
NS-II
NS-III
Fig. S1f
